# Image-guided spatial omics enhancement reveals hidden spatial microstructures

**DOI:** 10.1093/bioinformatics/btag596

**Published:** 2026-08-07

**Authors:** Jiahao Liu, Gongning Luo, Qiaoming Liu, Suyu Dong, Guohua Wang, Yuming Zhao

**Affiliations:** College of Computer and Control Engineering, Northeast Forestry University, Harbin, Heilongjiang, 150040, China; Faculty of Computing, Harbin Institute of Technology, Harbin, Heilongjiang, 150001, China; College of Artificial Intelligence, Henan University, Kaifeng, Henan, 475001, China; College of Computer and Control Engineering, Northeast Forestry University, Harbin, Heilongjiang, 150040, China; College of Computer and Control Engineering, Northeast Forestry University, Harbin, Heilongjiang, 150040, China; Faculty of Computing, Harbin Institute of Technology, Harbin, Heilongjiang, 150001, China; College of Computer and Control Engineering, Northeast Forestry University, Harbin, Heilongjiang, 150040, China

## Abstract

**Motivation:**

The rapid advancement of spatial omics is fundamentally hindered by the resolution gap between physical capture platforms and genuine biological microstructures, a challenge compounded by inherent data sparsity and noise. While current image-guided computational methods attempt to bridge this gap, they often lack the multi-modal flexibility, non-linear modeling, and scalability required for modern, whole-tissue datasets.

**Results:**

To address this, we introduce Bell, a modality-agnostic deep learning framework that reconstructs high-fidelity spatial microstructures by dynamically fusing histological images, spatial coordinates, and low-resolution molecular measurements via an adaptive attention mechanism. The study also presents mmBell, an extension utilizing a unified encoder structure to achieve cross-modal integration for increasingly complex multi-omics data. Systematically validated across over 10 spatial platforms and 20 datasets, Bell and mmBell consistently outperform state-of-the-art methods in resolution enhancement and noise suppression. Ultimately, this framework provides a highly robust, scalable solution for deeply deciphering complex spatial tissue organization.

**Availability and implementation:**

Bell is available from the GitHub repository.

## Graphical Abstract

**Graphical Abstract:**
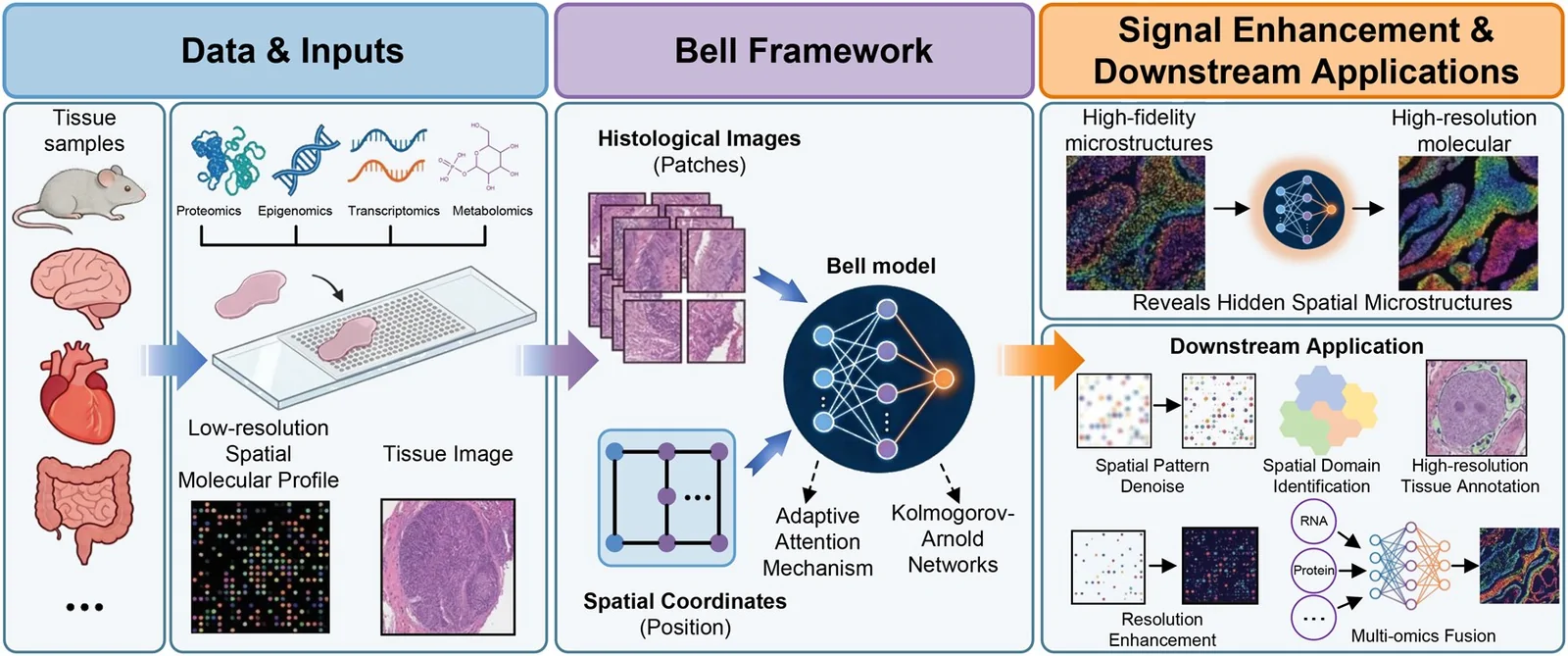

## 1 Introduction

In recent years, spatial omics technologies have advanced rapidly (Deng *et al.* 2022, Llorens-Bobadilla *et al.* 2023, Vandereyken *et al.* 2023, Zhang *et al.* 2023, Coleman *et al.* 2025); however, a central bottleneck continues to constrain their biological interpretability: a substantial gap remains between the physical capture resolution of current mainstream platforms and the scale of genuine biological structures. Most sequencing-based spatial platforms, such as Visium and related spot arrays (Rodriques *et al.* 2019), typically encompass dozens or even more heterogeneous cells within each capture unit (spot). Consequently, the measured molecular signals essentially represent spatial averages derived from mixed cellular populations, rather than originating from discrete, biologically well-defined cells or microstructural units. Concurrently, spatial sequencing data are frequently accompanied by high noise levels, pronounced sparsity, and frequent dropout events, which further obscure the underlying tissue architecture. Key biological features—such as microenvironmental niches, precise cellular boundaries, the spatial localization of rare cell populations, and subtle functional domains—are often mathematically homogenized, rendering them difficult to discern in downstream analyses. Thus, recovering hidden spatial microstructures from noisy, macroscopic measurements has emerged as one of the most pressing challenges in the field of spatial biology.

Imaging-based spatial technologies, such as Xenium and MERFISH (Chen *et al.* 2015, Janesick *et al.* 2023), while capable of achieving single-cell or even subcellular resolution for RNA detection, remain subject to multiple limitations, including a restricted panel of detectable genes, insufficient detection sensitivity, complex experimental and analytical workflows, and prohibitive overall costs (Huang *et al.* 2024). These factors collectively result in the continued predominance of sequencing-based spatial platforms for generating large-scale spatial omics datasets (Marx 2021, Jin *et al.* 2024). Meanwhile, an increasing number of novel platforms are being developed: for instance, the Spatial-DMT technique introduced by Deng *et al.* (2022) and Lee *et al.* (2025) enables spatially resolved detection of DNA methylation, while Spatial-Mux-seq, proposed by Guo *et al.* (2025), allows for the simultaneous in situ profiling of chromatin accessibility, two histone modifications, the transcriptome, and protein expression within the same tissue section. Nevertheless, these cutting-edge technologies remain in developmental stages, with their experimental protocols and computational analysis frameworks not yet fully matured. On one hand, constrained by underlying technical principles and detection strategies, current multimodal datasets commonly suffer from high noise, pronounced sparsity, missing data, and signal heterogeneity, which collectively impede the reliable extraction of genuine biological signals. On the other hand, discrepancies in sensitivity and resolution across modalities, coupled with systematic biases and batch effects introduced by joint detection workflows, together with the inherent heterogeneity of biological tissues, further exacerbate the challenges associated with data quality control and robust analytical interpretation (Long *et al.* 2024, Ge *et al.* 2025).

To bridge the gap between spatial resolution and biological structures, and to address the challenges posed by increasingly complex multi-omics data, a series of computational methods leveraging histological images to enhance spatial molecular data have been proposed. For instance, algorithms such as HisToGene (Pang *et al.* 2021), iStar (Zhang *et al.* 2024), STAGE (Li *et al.* 2024b), and XFuse (Bergenstråhle *et al.* 2022) attempt to combine histological morphological features with spatial coordinate information to infer gene expression maps at higher resolutions. Although these image-guided methods have improved spatial resolution to some extent, several key limitations persist in reconstructing genuine tissue microstructures. First, many methods rely on rigid or linear associations between morphological features and molecular expression, making them susceptible to staining artifacts or tissue morphological complexity, and hindering the characterization of complex, non-linear spatial boundaries in real biological tissues. Second, most methods are designed primarily for spatial transcriptomics data, lacking the architectural flexibility to accommodate emerging multimodal data such as spatial proteomics, epigenomics, and metabolomics. Furthermore, some methods rely on computationally intensive statistical models (e.g. Gaussian processes), whose scalability is significantly limited when faced with current high-density, whole-tissue section-scale datasets (Bergenstråhle *et al.* 2022, Li *et al.* 2024a, Zhang *et al.* 2024).

We present Bell, a unified computational framework specifically designed for spatial omics enhancement to systematically address the aforementioned issues. Bell is designed as a modality-agnostic, scalable deep learning system aimed at achieving spatial resolution enhancement, data denoising, and reliable reconstruction of hidden spatial microstructures through high-fidelity image-guided enhancement. By jointly integrating histological images, spatial coordinates, and low-resolution molecular measurements, the framework enables the model to establish connections between global tissue architecture and local fine-scale spatial patterns, thereby more accurately recovering true tissue microenvironment structures. At the architecture level, Bell introduces an adaptive attention mechanism to dynamically fuse image-derived morphological features with spatial position embeddings, allowing the model to comprehend overall tissue structure while precisely localizing fine-scale spatial dependencies. Bell is broadly applicable to various spatial omics data types, including spatial transcriptomics, spatial proteomics, spatial epigenomics, and spatial metabolomics. It can generate high-resolution molecular maps efficiently, significantly improving data quality and revealing tissue microstructures masked by noise and spatial averaging effects in the original data.

In a systematic evaluation encompassing more than ten spatial platforms and over twenty datasets, Bell consistently outperformed existing state-of-the-art methods in spatial resolution enhancement, noise suppression, and preservation of biological spatial topology. Using this framework, we successfully revealed spatial microstructures overlooked in original data, such as the highly compartmentalized structure within tonsil germinal centers. Considering that spatial biology is rapidly moving towards multi-modal integration, we further extended this framework by proposing mmBell. This model integrates data from different spatial omics modalities within a unified graph structure, achieving joint enhancement and synergistic interpretation of multi-omics information. Through cross-modal information fusion, mmBell not only further improves spatial resolution and signal stability but also enables more accurate identification of complex spatial domains and supports more reliable inference of developmental trajectories and regulatory networks. Overall, Bell and its multi-modal extension mmBell represent a unified computational framework tailored for future spatial omics research. By simultaneously achieving spatial resolution enhancement, data denoising, and multi-omics adaptability, this system can reveal true and fine tissue microstructures from complex and noisy spatial data, providing a powerful computational tool for deeply understanding the spatial organization principles of tissue biology.

## 2 Materials and methods

### 2.1 Overview of Bell

**Figure 1:**
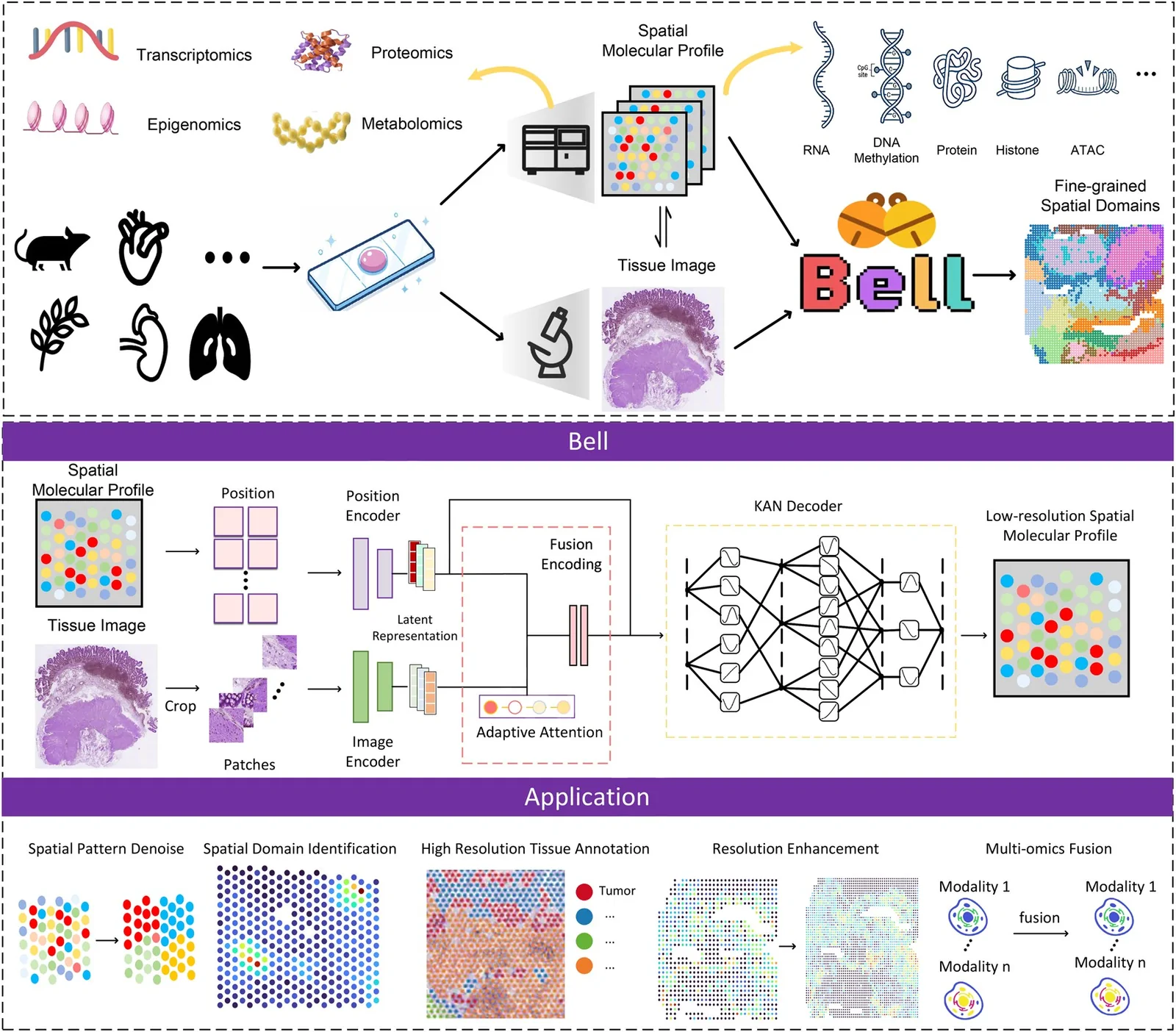
Overview of Bell and its applications. Bell is an efficient framework for high-resolution spatial omics generation, designed to unveil spatial microstructures obscured in original low-resolution data. It is highly versatile, adapting to diverse spatial platforms—including transcriptomics, proteomics, epigenomics, and metabolomics—and handling multi-dimensional molecular profiles such as RNA, DNA methylation, and ATAC. Methodologically, Bell integrates Kolmogorov-Arnold Networks (KAN) with an adaptive attention mechanism. By leveraging histology images and spatial coordinates, it infers latent spatial dependencies within low-resolution profiles to generate high-fidelity, high-resolution molecular maps. The enhanced molecular profiles enable a suite of downstream analyses, such as spatial pattern denoising, fine-grained spatial domain identification, high-resolution tissue annotation, and multi-omics integration.

Bell is a unified deep learning framework designed to enhance spatial omics data by reconstructing high-resolution molecular patterns from noisy and spatially coarse measurements (Fig. 1). The core idea of Bell is that tissue morphology and spatial topology contain rich structural information that can be used to recover latent molecular spatial organization. By jointly integrating histological image features, spatial location information, and measured molecular data, Bell can infer fine-grained spatial molecular patterns that are difficult to observe directly from raw data. In terms of architecture, Bell consists of four modules: gene filtering and preprocessing, feature embedding, fusion encoding, and molecular reconstruction. First, a spatial-information-based gene filtering strategy is applied, using Moran’s I to measure spatial autocorrelation and sampling from gene clusters with different autocorrelation levels to ensure diversity in spatial patterns. Next, feature embedding is performed: morphological features of image patches are extracted using a pretrained convolutional neural network, and spatial coordinates are embedded via a nonlinear encoder to capture positional relationships and spatial topological constraints. During the fusion stage, Bell introduces an adaptive attention mechanism to dynamically weight image features and integrate them with spatial embeddings, thereby highlighting key information and suppressing redundancy. The fused representation is modeled through a multilayer nonlinear structure based on Kolmogorov–Arnold Networks (KAN) (Liu *et al.* 2025), and the corresponding decoder reconstructs the molecular expression profile. The model is trained in a self-supervised manner, learning the intrinsic mapping between morphology, space, and molecular expression by minimizing the reconstruction error between predicted and observed values. After training, Bell can infer molecular expression at new spatial locations, enabling the reconstruction of high-resolution molecular maps while maintaining consistency with tissue morphology and spatial structure. Overall, Bell provides a general framework for recovering latent molecular structure from low-resolution, noisy spatial omics data, achieving simultaneous resolution enhancement, denoising, and structural reconstruction, thereby revealing spatial microstructures that are difficult to capture with traditional methods. A detailed schematic of the layer organization of Bell and its multimodal extension mmBell is provided in Fig. S8.

To evaluate the effectiveness of Bell, we conducted extensive comparative experiments with state-of-the-art methods on multiple real-world datasets (Table S1). Since high-resolution tasks lack ground-truth labels, we simulated the process of reconstructing high-resolution molecular profiles from low-resolution ones by randomly masking portions of the original data and then performing reconstruction. Our results demonstrate that Bell exhibits outstanding performance across multiple aspects, including generation quality, scalability, stability, and computational speed.

### 2.2 KAN layer

The recent emergence of Kolmogorov-Arnold Networks (KAN) has opened new opportunities in computational biology. Compared to traditional Multilayer Perceptrons (MLP), KANs demonstrate significant theoretical and technical advantages, particularly in function approximation efficiency, interpretability, and adaptability to scientific computing applications. In classic multilayer perceptrons, activation functions (e.g. ReLU, Sigmoid) are predefined and fixed, with only the weight matrices and bias parameters being adjusted during training. In contrast, Kolmogorov-Arnold Networks (Liu *et al.* 2025) abandon traditional linear weight transformations and instead employ learnable one-dimensional nonlinear functions as fundamental building blocks. These functions can dynamically adapt their shapes based on the data and approximate complex high-dimensional mappings through superposition and combination.

The core advantage of KANs in biological applications lies in their ability to approximate the intrinsic nonlinearity of biological systems with fewer assumptions and higher efficiency, while providing interpretability that is typically lacking in traditional deep learning approaches. We employ KAN layers to replace linear layers for better modeling of spatial relationships between spots and genes in spatial omics data. Multiple linear layers (Linear) can be defined as follows:


(1) $$\mathrm{Linear}(x)=\left(W_{l-1}{}^{\circ}\sigma {}^{\circ}W_{l-2}{}^{\circ}\sigma {}^{\circ}\cdots {}^{\circ}W_{1}{}^{\circ}\sigma {}^{\circ}W_{0}\right)x$$

where *l* denotes the layer number, σ represents the activation function, and *W* indicates the weight matrix.

An *l*-layer KAN can similarly be represented as a nested composition of multiple KAN layers:


(2) $$\mathrm{KAN}(x)=\left(\Phi_{l-1}{}^{\circ}\Phi_{l-2}{}^{\circ}\cdots {}^{\circ}\Phi_{1}{}^{\circ}\Phi_{0}\right)x$$

where $\Phi_{i}$ denotes the *i*th layer of the whole KAN network. The input and output of each KAN layer are $n_{in}$ and $n_{\mathrm{out}}$ dimensions, respectively, and Φ consists of $n_{in}\times n_{\mathrm{out}}$ learnable activation functions δ.


(3) $$\Phi =\{\delta_{q,p}\},p=1,2,\ldots ,n_{in},q=1,2,\ldots ,n_{\mathrm{out}}$$

The result of the computation of the KAN network from the *l*th layer to the l+1th layer can be expressed in matrix form as $x_{l+1}=\Phi_{l}x_{l}$.


(4) $$\begin{array}{c} \Phi_{l}=\left(\begin{array}{ccc} \delta_{l,1,1}(\cdot ) & \cdots & \delta_{l,1,n_{l}}(\cdot )\\ \vdots & \ddots & \vdots \\ \delta_{l,n_{l+1},1}(\cdot ) & \cdots & \delta_{l,n_{l+1},n_{l}}(\cdot ) \end{array}\right) \end{array}$$

### 2.3 Data processing

Bell uses a set of genes with significant spatial characteristics as model input. Specifically, gene-level preprocessing is first performed on the original expression matrix. Since mitochondrial genes often reflect cellular metabolic states or technical noise rather than meaningful spatial structure, all mitochondrial genes are removed at the beginning of the analysis. Next, to ensure the stability and reliability of subsequent spatial statistics, genes expressed in fewer than 30 spatial spots are further filtered out, thereby reducing biases caused by sparse expression. After this initial filtering, Moran’s I index is calculated for each remaining gene. This statistic provides a quantitative measure of spatial autocorrelation, enabling genes to be characterized in terms of whether and to what extent they exhibit spatial structure. Instead of applying a fixed threshold to select genes, the Moran’s I scores of all genes are then treated as a one-dimensional feature and clustered using the DBSCAN algorithm. The use of DBSCAN allows genes with similar levels of spatial autocorrelation to be automatically grouped based on the density distribution of Moran’s I scores, without requiring a predefined number of clusters, while also identifying potential outlier genes. Finally, within each Moran’s I cluster, approximately 30% of the genes are randomly sampled and combined to form the final gene list. This strategy ensures that genes representing different degrees of spatial characteristics are adequately covered, while preventing genes with extremely high or low Moran’s I values from dominating the input, thereby enhancing the diversity and representativeness of the model input.

### 2.4 Feature embedding

In prior work, histological image features have often been integrated with molecular profiles using neural networks, typically relying on large pre-trained backbones that are resource-intensive. While image features are informative, we argue that positional information is more critical for high-resolution generation, given the strong local correlations in molecular patterns—hence the popularity of graph neural networks in spatial multi-omics. Using image features simplifies inference, whereas molecular-profile-based methods face missing data issues and often rely on computationally heavy Gaussian processes. Therefore, Bell focuses on bridging image and molecular features with neural networks, while incorporating topological semantics from spatial relationships to constrain optimization.

Given the image and its spatially matched molecular profiles, we cropped 32 × 32 patches centered on each spot from the image as its visual input. We employed a pre-trained ResNet18 as the feature extractor to encode all patches into 512-dimensional latent embeddings. To facilitate subsequent fusion encoding, we projected these latent embeddings to 128 dimensions using a linear layer with Dropout. For spatial positional features, we directly used the x and y pixel coordinates of each spot in the image as positional inputs, which were then projected to 128 dimensions through a KAN layer with Batch Normalization.

### 2.5 Fusion encoding

Both images and genes contain substantial redundant components (identical feature patterns) in their characteristics, and the projection operation in feature embedding has already performed preliminary dimensionality reduction on the data. However, not all latent embeddings of images contribute to fusion with the latent embeddings of genes. To boost the ability of the model to learn image and gene features to better perform the high-resolution generation task, we employ a 128-dimensional adaptive attention mechanism to help filter image features. This adaptive attention is initialized to all ones before model training and is jointly optimized during learning through the loss function and the model. We use two stacked KAN layers to learn the features fused from images and spatial positions. Additionally, to prevent spatial information loss, an extra residual branch directly transmits the latent encoding of spatial positions into the fused encoded features. This computational process can be expressed by the following formula:


(5) $$\varphi =\text{FusionEncoding}(\theta \times \eta_{\mathrm{img}}+\eta_{\mathrm{pos}})+\eta_{\mathrm{pos}}$$

where φ is the fusion-coded feature, $\eta_{\mathrm{img}}$ and $\eta_{\mathrm{pos}}$ are the 128-dimensional image latent representation and spatial location latent representation obtained after feature embedding, respectively, and θ is the adaptive attention.

### 2.6 Decoder and model optimization

To reconstruct the complete molecular profile from the fused encoded features φ, we employ a decoder composed of four stacked KAN layers. The first three KAN layers maintain a 128-dimensional space for feature learning, while the final KAN layer aligns the features from 128 dimensions to the target dimensionality of the molecular profile to be generated.

After obtaining the reconstructed molecular profile through the above process, we optimize the model by minimizing the reconstruction loss between the generated output and the ground-truth molecular profile used for training. The computation of the specific loss function is as follows:


(6) $$\ell_{\mathrm{Bell}}=\frac{1}{n}\sum_{j=1}^{n}{\left(v_{j}-{\hat{v}}_{j}\right)}^{2}$$

where *v* and $\hat{v}$ are the reconstructed and true molecular profiles, respectively, and n is the number of spots in the molecular profile.

We used mean squared error (MSE) as the primary optimization objective because it directly preserves quantitative agreement between reconstructed and measured molecular profiles, which is essential for downstream analyses that depend on molecular abundance values. Although SSIM-like structural losses can be useful in natural image reconstruction, direct application to spatial omics is not straightforward because molecular profiles are high-dimensional, sparse, zero-inflated, and feature-dependent. Strong structural penalties may also oversmooth local molecular transitions and produce visually coherent but biologically inaccurate spatial patterns. In the current implementation, Bell encodes spatial coherence through coordinate embeddings, image-guided feature fusion, and adaptive attention, while MSE preserves quantitative molecular fidelity. Topology-aware losses that explicitly preserve molecular boundaries without oversmoothing localized biological transitions remain a useful direction for future extensions.

Since the entire method is unsupervised and the amount of data available when learning on each sample is very small. Training directly on a single sample will result in a model that fails to converge, and we perform a number of processes to increase the richness of the data. First, we randomize the order of the spatial locations of the input data during training. Second, in the early stage of the training phase, we dynamically sample small batches from the original data for training, and each time we only sample 20%–40% of all the data for training. At the later stage of training, we then use all the data to fine-tune the model. Specifically, we judge the stage of training based on the progress of epoch, and in our default setting when the ratio of epoch to maximum epoch is less than 90%, the model is trained only with dynamic small batches. When the ratio is greater than 90%, all data are used for fine-tuning.

Users can adjust the hyper-parameters of Bell according to the actual situation of different data to achieve better results. We also provide a set of default parameter settings for Bell for general processing. For all data sets, Bell uses 0.001 as the learning rate and Adam as the optimizer. For the recovery task, the epoch is set to 500. And for high-resolution generation, the epoch is set to 3000. Detailed architecture and training settings for Bell and mmBell are summarized in Table S3 and Fig. S8, and the robustness of key training choices is evaluated in Fig. S2.

### 2.7 mmBell

To handle multimodal data, we extend Bell to mmBell. Overall, mmBell adopts the same basic setup as Bell. In general, mmBell can simultaneously process any number of modalities. For ease of explanation, we illustrate the case with two modalities as an example. mmBell employs a shared position encoder, image encoder, and fusion encoding module to process positional data (Position1, Position2) and image data (Patches1, Patches2) from two different modalities. It then uses two independent decoders to separately process the data from each modality. After obtaining the reconstructed molecular profiles for both modalities, we compute the reconstruction loss for each modality individually and then jointly optimize the entire mmBell model (including shared components and independent decoders). The overall computational workflow is as follows:


(7) $$\ell_{\mathrm{mmBell}}=\frac{1}{n}\sum_{j=1}^{n}{\left(v_{j}^{1}-{\hat{v}}_{j}^{1}\right)}^{2}+\frac{1}{m}\sum_{j=1}^{m}{\left(v_{j}^{2}-{\hat{v}}_{j}^{2}\right)}^{2}$$

where *v* and $\hat{v}$ are the reconstructed molecular profile and the true molecular profile, respectively, and the superscripts of 1 and 2 represent the different modalities, respectively. *n* is the number of spots in the molecular profile of modality 1, and *m* is the number of spots in the molecular profile of modality 2.

In the current implementation, the modality-specific reconstruction losses are equally weighted after each modality has been independently preprocessed and normalized. This strategy reduces scale differences between molecular layers, and the use of modality-specific decoders prevents one reconstruction head from directly fitting the noise distribution of another modality. Nevertheless, modality imbalance can still affect the shared latent representation when one modality has substantially higher noise, sparsity, or dropout. Uncertainty-aware, adaptive, or noise-aware modality weighting may further improve robustness in future versions of mmBell, especially for datasets in which one molecular layer is much noisier than the others.

### 2.8 Biological variation and score inter-gene correlation calculation

We employ the classic mean-variance modeling approach from single-cell analysis to calculate the biological variation score. First, we compute the mean molecular level and total variance of each gene across all spots. Then, we use LOWESS local regression to fit the dependence between the mean molecular and variance, taking the fitted values as estimates of technical noise. Next, we subtract the technical variance from the total variance to obtain the biological variation score, which reflects true biological differences. This method effectively distinguishes technical noise from biological signals, providing a basis for subsequent screening of highly variable genes. Based on the computed biological variation scores, we reorder the genes in the data from highest to lowest and select the top 60 genes from both the original and reconstructed molecular profiles. We then cluster these genes to group those with similar molecular levels together. Finally, we calculate the Pearson correlation coefficients between genes to determine their pairwise correlations.

### 2.9 Dataset processing

For a fair quantitative comparison, we performed a uniform and simple processing of all datasets. Specifically, for samples with more than 3000 features we selected their top 3000 highly variable features. Otherwise, all features were retained. We used the *highly_variable_genes* function in the scanpy (Wolf *et al.* 2018) package to set the flavor to *seurat_v3* to filter the highly variable genes, and normalized the gene data with the *normalize_total* and *log*1*p* functions. Some special treatments for particular datasets are as follows:

**Spatial-CUT&Tag and ATAC-seq for mouse embryos dataset:** Spatial-CUT&Tag obtained single-cell epigenomes by identifying 20-micrometer pixels containing only one nucleus using late immunofluorescence imaging. Of these, both histone and ATAC data were stored using fragments files. We used the R package of ArchR to add the mouse genome to the current ArchR project via addArchRGenome(“mm10”), transformed the raw fragments file into a matrix of spot×gene, and then processed it through the same process as the other data.

### 2.10 High-resolution experiment

In high-resolution experiments, different methods aim to enhance the original molecular profile by a factor of *N*. We employ a grid sampling procedure to define new spatial locations that require high-resolution inference while preserving the tissue footprint. Let the original spatial coordinate set be $S={\left\{s_{i}\right\}}_{i=1}^{n}$, where $s_{i}\in R^{2}$ denotes the two-dimensional coordinate of the *i*th measured spot. We first calculate the nearest-neighbor distance for each original spot and use the average value as a tissue-support threshold:


(8) $$\bar{d}=\frac{1}{n}\sum_{i=1}^{n}\underset{k\neq i}{\text{min}}\|s_{i}-s_{k}{\|}_{2}.$$

For an *N*-fold high-resolution generation task, we generate a regular candidate grid *G* over the bounding box of the original tissue region at approximately *N*-fold higher spatial density. Because real-world samples may contain tissue voids, unsequenced regions, or irregular boundaries, not every candidate grid point should be retained. A candidate point g∈G is kept only if it is close enough to the measured tissue support:


(9) $$\underset{s_{i}\in S}{\min}\|g-s_{i}{\|}_{2}\leq \bar{d}.$$

This filtering step removes candidate points located in background regions, tissue voids, or outside irregular tissue boundaries, thereby preserving the overall tissue shape while producing a high-resolution coordinate set with approximately *N* times the original spot density. For each retained candidate location, Bell extracts the corresponding image patch, encodes its spatial coordinate, and predicts the molecular profile using the trained model. This grid-generation procedure is used for high-resolution generation experiments such as 4× or 8× enhancement. It is not required for recovery or denoising benchmarks in which the task is to reconstruct masked, downsampled, or otherwise predefined target coordinates.

## 3 Results

### 3.1 Bell can restore spatial transcriptomic biological signals efficiently and precisely

**Figure 2:**
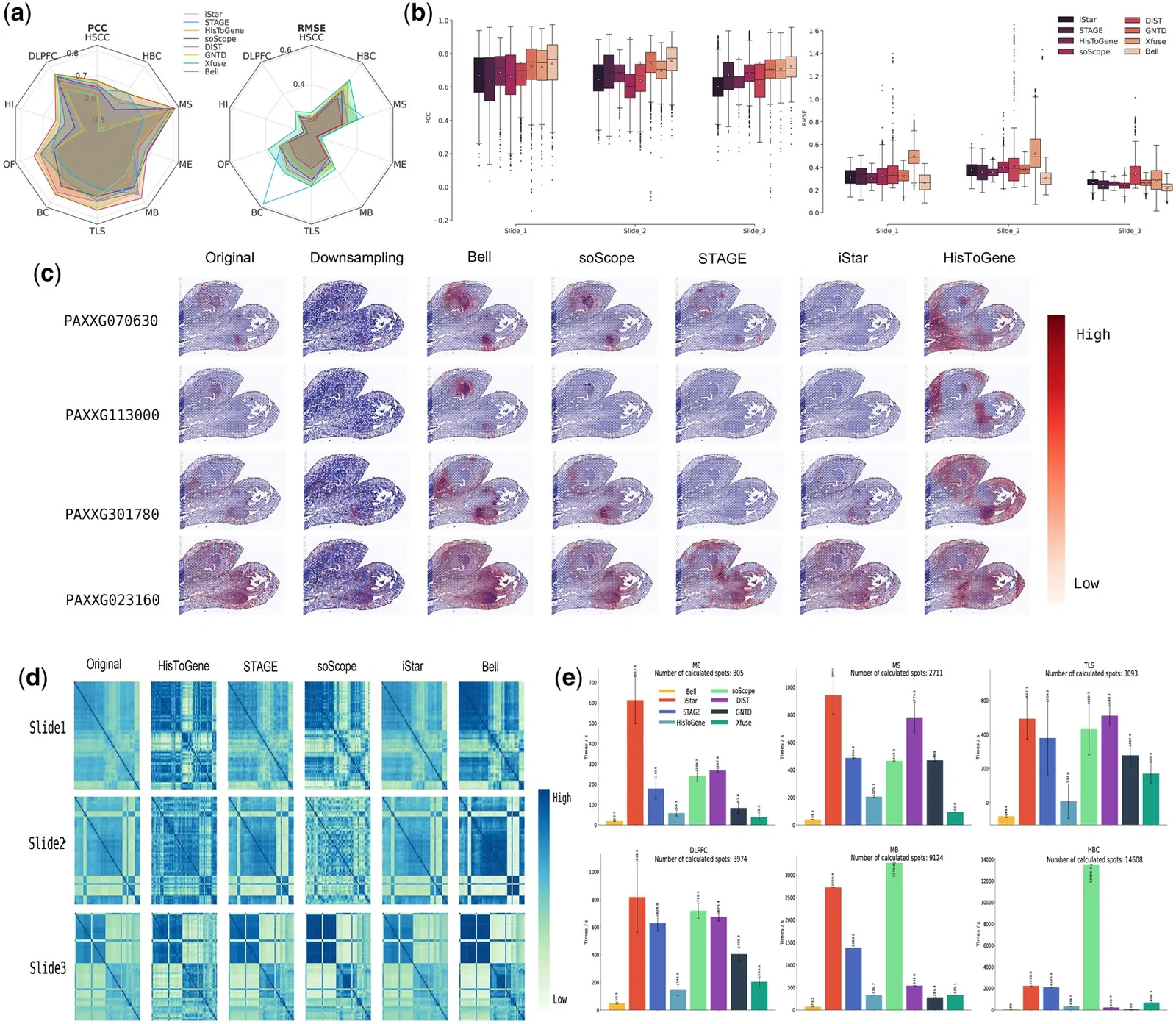
Evaluation of Bell across multiple spatial transcriptomics platforms. (a) Comparison of Pearson correlation coefficients (PCC) and root mean squared error (RMSE) between Bell and other methods on 10 spatial transcriptomics datasets. (b) PCC and RMSE comparison between Bell and other methods across three sections of an orchid floral (OF) dataset generated by Visium. (c) Spatial molecular patterns of four MADS-box genes associated with orchid development in Slide_1. ‘Original’ and ‘Downsampling’ represent raw data and training data, respectively, while other panels show reconstructed results from different methods. (d) Correlation matrices of the top 60 most biologically variable genes across three sections of the orchid developmental dataset. (e) Computational time of different methods across six datasets with different input sizes: ME, MS, TLS, DLPFC, MB, and HBC. Spot numbers are labeled in each subpanel. This panel evaluates computational efficiency rather than reconstruction accuracy.

To evaluate whether Bell can accurately recover spatial molecular signals from noisy and incomplete measurements, we systematically benchmarked its reconstruction performance across 10 spatial transcriptomics datasets. These datasets were generated from multiple platforms, including Visium, DBiT-seq, Stereo-seq, and Xenium. These platforms represent a broad range of spatial resolutions and measurement strategies, providing a comprehensive testbed for assessing the robustness and generalization capability of the framework. For each sample in every dataset, 50% of the spots in the original data were randomly masked, and different algorithms were applied to reconstruct the original molecular expression profiles. This setup preserves the severe zero-inflation or technical dropout effects commonly observed in sequencing technologies, simulating the extreme sparsity caused by insufficient sequencing depth and thereby providing a realistic test of denoising and interpolation capability. Reconstruction accuracy was quantified by calculating the Pearson correlation coefficient (PCC) and root mean squared error (RMSE) between the reconstructed expression profiles and the original measurements. As shown in Fig. 2a, Bell consistently achieved the highest PCC values and the lowest RMSE across all datasets, indicating that it most faithfully recovered the underlying spatial molecular patterns.

We then examined reconstruction performance on a representative orchid developmental spatial transcriptomics dataset generated using the Visium platform (Liu *et al.* 2022). This dataset contains three tissue sections (slides 1–3), each comprising approximately 2500 spatial spots. Bell consistently outperformed competing methods across all three sections (Fig. 2b), demonstrating stable reconstruction accuracy across biological replicates. To further illustrate the biological relevance of the reconstructed signals, we visualized several developmental regulators belonging to the MADS-box gene family in slide 1 (Fig. 2c). These genes function as master regulators of plant organ formation and exhibit well-defined spatial expression domains during floral development. In the original data, sequencing noise partially obscured these spatial patterns, while downsampling further disrupted their intrinsic structure. Among the comparison methods, iStar (Zhang *et al.* 2024) was able to recover genes with strong spatial signals, such as PAXXG301780 and PAXXG023160, but showed limited performance in regions surrounding the gynostemium tissue. soScope (Li *et al.* 2024a) integrates histological images and molecular information and produced relatively stable reconstructions in the labellum and gynostemium regions; however, it struggled to recover weakly expressed genes, such as PAXXG113000 located near the tip of the gynostemium. In contrast, Bell consistently recovered coherent spatial patterns even for low-abundance genes, suggesting that the framework can effectively recover subtle biological signals that are easily masked by noise. Because biological interpretation often depends on coordinated gene regulation rather than individual genes alone, we further evaluated whether the reconstructed data preserved gene–gene relationships. For each tissue section, we selected the top 60 highly variable genes and computed their correlation matrices based on both the original and reconstructed data (Fig. 2d). Most methods partially recovered the overall correlation structure, while neural-network-based approaches tended to produce stronger correlation patterns due to their inherent denoising effects. Notably, Bell reconstructed the most consistent and biologically coherent correlation structure while preserving clear spatial expression boundaries, suggesting that the recovered signals reflect genuine regulatory relationships rather than artificial smoothing.

We further evaluated Bell on a human breast cancer spatial transcriptomics dataset generated using the Xenium platform (Janesick *et al.* 2023). Due to the large size of the tissue section, we selected a predefined ROI containing 14 608 spatial spots for visualization. The ROI was chosen prior to model reconstruction because it contains sufficient tissue coverage, high spot density, minimal imaging artifacts, and clear tumor boundary structures. We examined several established marker genes associated with tumor progression and myoepithelial cell characteristics, including TACSTD2 and FOXA1 (markers of tumor invasion and metastasis), as well as ACTA2 and KRT15 (myoepithelial markers). After downsampling, the ACTA2 expression domain along the tumor boundary became noticeably blurred in the observed data. Although STAGE (Li *et al.* 2024b) and soScope (Li *et al.* 2024a) partially recovered the spatial pattern, their reconstructed results remained diffuse and fragmented. In contrast, Bell precisely restored the clear spatial boundary of ACTA2 expression and generated expression maps that were spatially more coherent than those derived from the original measurements (Fig. S1), highlighting its ability to reconstruct fine-scale spatial structures within complex tumor microenvironments.

Finally, we compared the computational efficiency of these methods across six datasets with varying numbers of spatial spots: ME, MS, TLS, DLPFC, MB, and HBC. The dataset identities and platforms are summarized in Table S1, and the corresponding spot numbers are labeled directly in Fig. 2e. These datasets span input sizes from 805 to 14 608 spots, allowing us to evaluate how the running time of Bell and competing methods changes with dataset scale. Unlike the other panels in Fig. 2, which mainly assess reconstruction accuracy, Fig. 2e focuses on computational efficiency. For high-resolution generation, runtime is strongly related to the number of input spatial spots. As expected, the computational cost of all methods increased with dataset size. However, methods relying on Gaussian process models, such as soScope (Li *et al.* 2024a), exhibited particularly steep increases in runtime as the data volume grew. Approaches such as STAGE and iStar showed approximately linear scaling but still required 6–20 minutes to process datasets containing approximately 800 spots. In contrast, benefiting from an efficient optimization strategy and a highly parallelizable architecture, Bell maintained a runtime of under two minutes across all datasets. Additional CPU and GPU memory measurements, including the GPU memory curve across spot numbers, are provided in Table S2 and Fig. S3. Overall, these results demonstrate that Bell can accurately reconstruct spatial molecular maps from incomplete and noisy spatial transcriptomics data while preserving biologically meaningful spatial organization and regulatory relationships. This capability provides a critical foundation for recovering hidden spatial microstructures that are often obscured by technical noise and spatial averaging in current spatial omics technologies.

### 3.2 Bell can enhance and denoise spatial omics structural domains

To evaluate whether Bell can generalize beyond spatial transcriptomics, we systematically assessed its performance across multiple spatial omics modalities. Specifically, we analyzed seven datasets, including four spatial proteomics datasets, one spatial metabolomics dataset, and two spatial epigenomics datasets comprising ATAC-seq and histone modification profiles. Because spatial aggregation alone cannot fully reproduce sequencing-like degradation, we followed the Spotless (Sang-Aram *et al.* 2024) experimental design to construct a realistic benchmark. For each dataset, we first simulated realistic low-resolution inputs by aggregating the high-resolution expression matrix into low-resolution spots using a fixed spatial grid. We then added sequencing-like degradation effects to the aggregated count data, including library-size downsampling, negative-binomial overdispersion, dropout, and ambient RNA contamination. Thus, the input data were not simply spatial averages of the ground-truth values. Instead, they represented degraded low-resolution measurements that more closely resemble noisy real-world spatial omics data.

**Figure 3:**
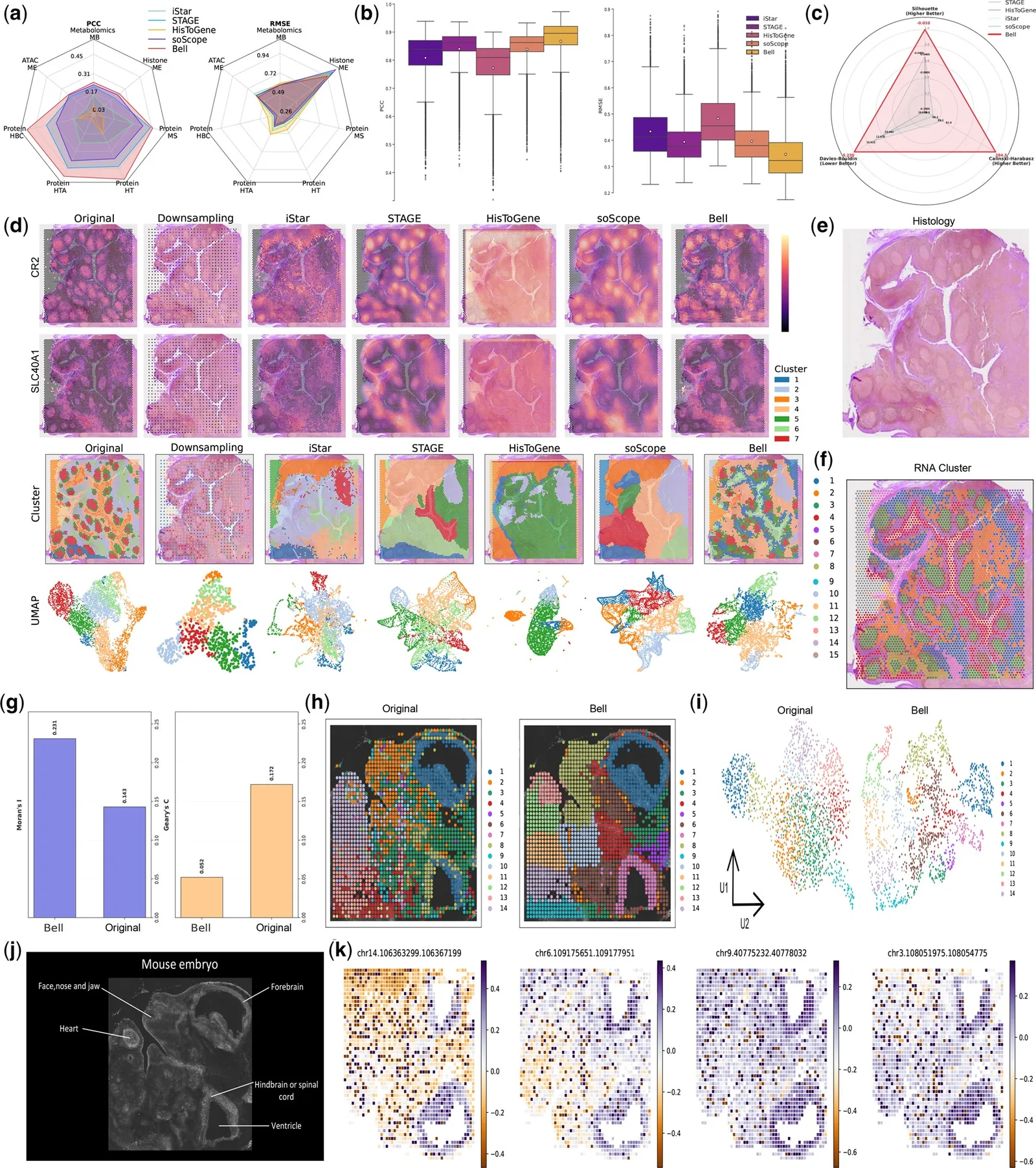
Evaluation of Bell across multiple spatial omics platforms. (a) Comparison of pearson correlation coefficients (PCC) and root mean squared error (RMSE) between Bell and other methods on seven spatial omics datasets, including spatial proteomics, spatial epigenomics, and spatial metabolomics data. (b) PCC and RMSE comparison between Bell and other methods on a human tonsil spatial proteomics dataset (Protein HT) generated by Visium CytAssist. (c) Silhouette, Calinski-Harabasz, and Davies-Bouldin scores for the clustering results of the human tonsil spatial proteomics data (Protein HT). (d) Visualization of spatially patterned protein molecular profiles, cluster, and UMAP in the human tonsil dataset (Protein HT). ‘Original’ and ‘Downsampling’ represent raw data and training data, respectively, while other panels show reconstructed results from different methods. (e) Histology image of human tonsil dataset (Protein HT). (f) Spatial transcriptomics clustering results of human tonsil dataset (Protein HT). (g) Spatial autocorrelation results on the human tonsil dataset (Protein HT). (h) Clustering results of original data and Bell in mouse embryonic methylation data. i. UMAP plot of mouse embryonic methylation data. (j) Brightfield images of mouse embryos and partial tissue annotations. (k) Spatial variably methylated regions of mouse embryonic methylation data.

Across all datasets, Bell consistently achieved the best reconstruction accuracy among all compared methods. It produced the highest Pearson correlation coefficient (PCC) and the lowest root mean squared error (RMSE) (Fig. 3a). These results indicate that Bell is not limited to transcriptomic measurements, but can robustly enhance spatial molecular signals across diverse omics modalities. We then performed an in-depth analysis of a human tonsil spatial proteomics dataset generated by Visium CytAssist, which also provides paired spatial transcriptomic data. In the quantitative comparison, Bell outperformed existing methods (Fig. 3b). We used the Wilcoxon rank-sum test to identify the top 20 differentially expressed genes in each cluster. We then visualized representative markers of key immune cell populations, including CR2, a surface receptor of mature B cells and follicular dendritic cells, and SLC40A1, a macrophage-, myeloid-, and iron-handling-related marker (Fig. 3d). In the original data, both proteins showed clear germinal-center-enriched spatial structures. After realistic downsampling, the data became sparse, blurred, and highly noisy. This phenomenon arises because the diameter of a capture unit, or spot, usually exceeds that of a single cell. During sampling, signals from multiple cells are physically averaged, which masks fine-scale spatial heterogeneity. Most existing reconstruction methods failed to recover these spatial patterns. STAGE and soScope partially restored the spatial signals, but showed clear boundary blurring and detail distortion. In contrast, Bell almost completely reconstructed these patterns. Spatial autocorrelation analysis further confirmed that Bell markedly improved Moran’s I and Geary’s C statistics (Fig. 3g). This indicates that Bell restored spatial dependency structures disrupted by downsampling and enhanced intrinsic spatial signals in the data.

Next, we uniformly clustered the results from all methods using the mclust algorithm and divided the original and reconstructed profiles into 15 clusters. In the original dataset (Fig. 3d, third row), cluster 7 clearly corresponded to the germinal center, and the clusters showed well-organized distributions in UMAP space. However, most of these spatial features were lost after realistic downsampling and noise addition. Among all methods, only Bell recovered part of the germinal center structure. Notably, Bell also successfully recovered the mantle zone within the tonsillar microanatomy, corresponding to clusters 7 and 6. This result was highly consistent with clustering results derived from spatial transcriptomics (Fig. 3e and f). These findings highlight Bell’s ability to recover biologically meaningful spatial structures from highly degraded measurements.

Because many spatial omics datasets lack ground-truth spatial annotations, we further performed quantitative comparisons using three widely adopted unsupervised clustering metrics. The silhouette score evaluates cluster compactness and separation. The Calinski-Harabasz Index measures the ratio of between-cluster variance to within-cluster variance. The Davies-Bouldin Index assesses similarity between clusters. Bell achieved the best performance across all three metrics (Fig. 3c), indicating clearer cluster structure, stronger inter-cluster discrimination, and greater stability than competing methods.

Importantly, Bell was also shown to be effective for emerging spatial omics technologies that typically exhibit substantial technical noise. Recently, Deng and colleagues introduced Spatial-DMT, the first technology capable of detecting spatially resolved DNA cytosine methylation. However, such early-stage platforms often generate highly noisy datasets, and dedicated analysis tools remain limited. In a mouse embryo spatial methylation dataset generated by Spatial-DMT, direct clustering of the raw data could only separate coarse anatomical regions such as the forebrain and hindbrain (Fig. 3h and j), with substantial overlap remaining between clusters. After Bell denoising, finer anatomical structures—including the heart and facial regions—became clearly distinguishable. Consistent with these observations, UMAP visualization showed markedly improved cluster separation after Bell processing (Fig. 3i). For example, clusters representing facial structures that were previously inseparable in the raw data became clearly resolved after Bell enhancement. Analysis of spatially variable methylated regions further supported the accuracy of these reconstructed spatial patterns (Fig. 3k). In summary, these results demonstrate that Bell can robustly enhance spatial structural signals across a wide range of spatial omics modalities—including proteomics, metabolomics, epigenomics, and emerging spatial methylation technologies—thereby providing a unified framework for recovering biologically meaningful spatial microstructures from noisy and low-resolution spatial measurements.

### 3.3 Bell accurately reconstructs real high-resolution spatial molecular patterns

**Figure 4:**
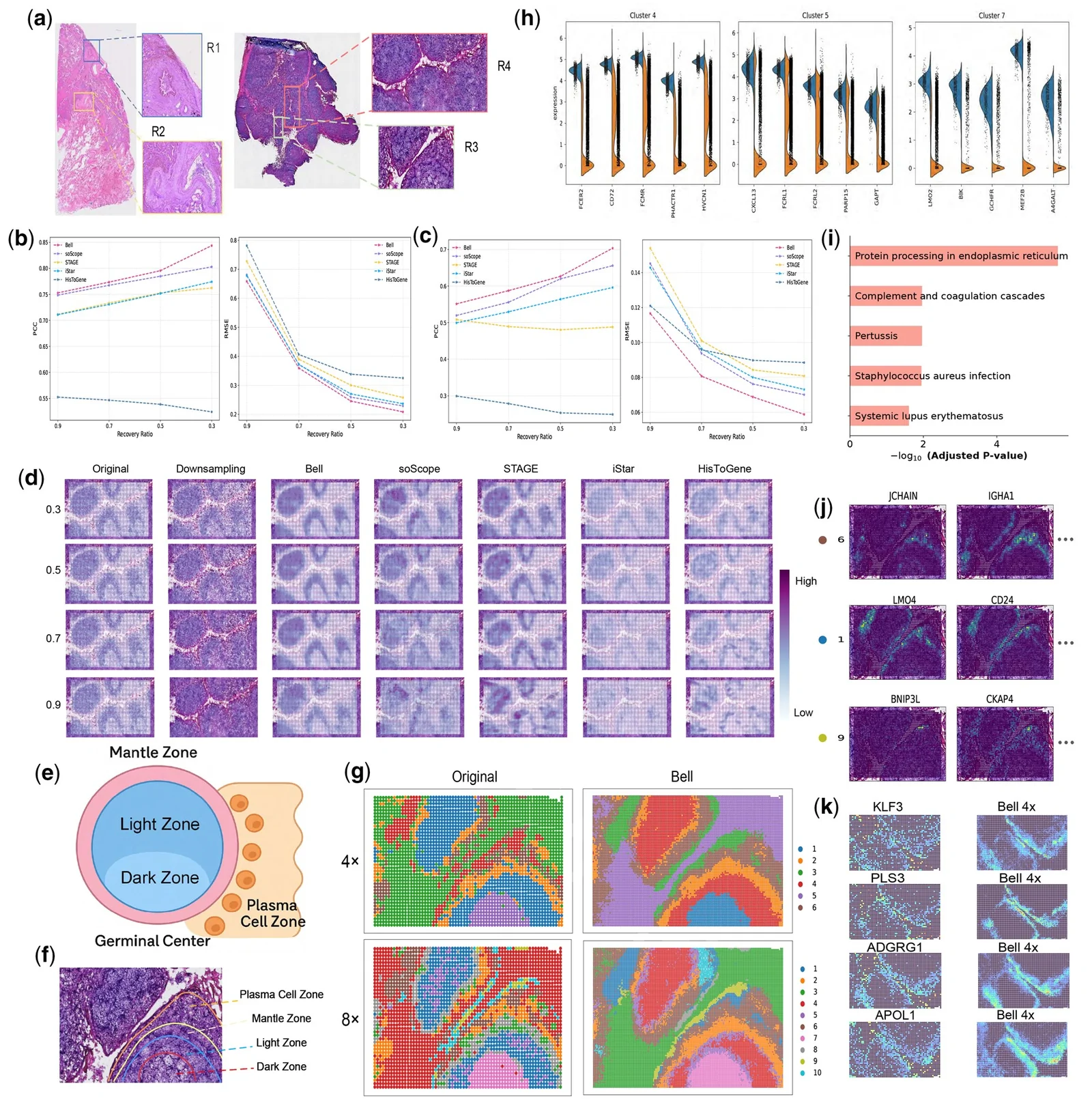
Evaluation of Bell at different resolutions and high-resolution tissue structure annotation. (a) Region cropping and corresponding tissue images of high-resolution Visium HD data from human lung cancer (HD HLC) and human tonsil (HD HT). (b) Comparison of Pearson correlation coefficients and root mean squared error (RMSE) between Bell and other methods on human lung cancer data at different resolutions. (c) Comparison of Pearson correlation coefficients and root mean squared error (RMSE) between Bell and other methods on human tonsil data at different resolutions. (d) Spatial pattern visualization of the FCRL1 gene in region R4 of the human tonsil dataset. (e) Germinal center structural composition. (f) Manual annotation of R3 region. (g) Clustering comparison between the original data and Bell data in region R4 of the human tonsil dataset under different cluster numbers. (h) Differential analysis results for specific clusters. (i) KEGG enrichment analysis results for clusters (1, 6, 9). (j) Differentially expressed genes in clusters (1, 6, 9). (k) Genes related to tissue structure and their enhanced expression of the human tonsil dataset.

To more stringently assess whether Bell can recover genuine high-resolution spatial molecular patterns, rather than artifacts introduced by simulated downsampling, we selected one human lung cancer dataset and one human tonsil dataset generated by high-resolution Visium HD (Oliveira *et al.* 2025). These datasets were used to evaluate Bell across different spatial resolutions. Visium HD provides near-single-cell spatial resolution (approximately 2 μm), so its measured expression profiles can serve as a proxy for the true high-resolution molecular landscape. Because whole-section inference is difficult for some competing methods, we extracted four regions of interest, R1, R2, R3, and R4, from these datasets and benchmarked all methods following the Spotless (Sang-Aram *et al.* 2024) experimental setting described in the previous section. The original Visium HD measurements were used as the reference high-resolution spatial transcriptome. We then downsampled the data to multiple spatial resolution levels corresponding to 30%, 50%, 70%, and 90% reductions in capture spot density (Fig. 4a). The downsampled data were used to train Bell and the competing methods, which were required to reconstruct the original high-resolution spatial expression patterns.

Across all downsampling levels, Bell consistently achieved the highest PCC and the lowest RMSE among all evaluated methods (Fig. 4b and c). These results indicate that Bell more faithfully recovers the true high-resolution spatial molecular landscape, even in the challenging setting where 90% of capture spots are removed. Bell therefore showed strong stability and robustness in this complex real-data benchmark. We further examined how each method recovered the actual spatial structure within these regions. For example, in region R4 of the human tonsil dataset, FCRL1 expression generally marks regions enriched for follicular B cells, mature B cells, or germinal-center-associated B cells (Fig. 4d). As the available spatial resolution decreased, the reconstructions generated by most methods became increasingly blurred. In particular, iStar and soScope almost completely failed when reconstructing from highly sparse inputs with a 90% reduction in capture spots. STAGE and HisToGene recovered some regional features, but most fine spatial details were lost, resulting in diffuse or fragmented expression patterns. In contrast, Bell preserved sharper spatial boundaries and more coherent spatial domains, with reconstructed gene expression concentrated around the germinal center. This pattern closely matched the original high-resolution expression landscape. Importantly, the reconstructed pattern was not simply a smoothed interpolation of the input signal. Instead, Bell recovered fine spatial structures that were consistent with both the underlying tissue morphology and the original high-resolution molecular landscape. Further clustering and UMAP analyses of R4 also showed that Bell produced clearer and more continuous cluster structures than the other methods (Fig. S5a–c).

Overall, these results demonstrate that Bell can reliably reconstruct genuine high-resolution spatial molecular patterns from degraded measurements. By leveraging complementary information from tissue morphology, spatial topology, and molecular profiles, Bell effectively reverses the spatial averaging effect introduced by low-resolution measurements, enabling accurate recovery of spatial gene expression landscapes that closely match the original high-resolution data.

### 3.4 Bell can enhance resolution for precise analysis of germinal center substructures

The limited resolution of current spatial sequencing technologies often obscures fine tissue microarchitecture. Signals captured from each capture spot typically represent a mixture of multiple heterogeneous cells, leading to blurred spatial patterns and fragmented structural boundaries. As a result, biologically meaningful microdomains within tissues may remain hidden in the raw data. To determine whether Bell can recover such latent spatial organization, we evaluated its ability to reconstruct high-resolution spatial patterns and reveal previously indistinguishable tissue microstructures.

To directly test whether Bell’s reconstructed patterns correspond to biologically meaningful tissue microstructures, we focused on the germinal center structure in a human tonsil Visium HD dataset (Oliveira *et al.* 2025). Germinal centers represent highly organized immune microenvironments composed of several functional subregions, including the dark zone, light zone, mantle zone, and plasma cell differentiation region. However, due to the spatial averaging effect, the boundaries between these compartments often appear fragmented or poorly defined in the original data. We analyzed region R4 of the tonsil dataset, which contains 2783 capture spots, and performed clustering analysis using mclust (Fraley *et al.* 2012) on both the original data and the Bell-enhanced reconstructed data (Fig. 4e–g). In the original dataset, clusters roughly corresponding to the dark and light zones could be identified, but the peripheral regions of the germinal center exhibited fragmented and diffuse structures. After applying Bell for 4× and 8× spatial enhancement, previously diffuse regions became spatially coherent, revealing clearer domain boundaries. Notably, the 8× enhancement reconstructed a well-organized peripheral structure that was not apparent in the original data, suggesting the emergence of additional spatial domains. Differential expression analysis of the ten clusters identified in the 8× enhanced data further supported the biological relevance of these newly resolved structures (Fig. 4h). Genes enriched in cluster 7 included BIK, LMO2, and MEF2B, which are associated with apoptosis during affinity selection and early germinal center formation, consistent with the phenotype of dark zone B cells. Clusters 4 and 5 exhibited sustained expression of FCER2 and CXCL13, suggesting that these B-cell populations are undergoing selection and interacting with follicular dendritic cells and follicular helper T cells, characteristic of the light zone. Importantly, Bell also revealed additional clusters (clusters 1, 6, and 9) that could not be clearly distinguished in the original data. These clusters showed high expression of core plasma cell markers such as PRDM1, IGHA1, and XBP1, as well as classical plasma cell markers including JCHAIN and SDC1 (Fig. 4j). Their spatial localization around the periphery of the germinal center suggests the presence of a plasma cell differentiation zone, representing the output region of the germinal center reaction where B cells differentiate into antibody-secreting cells. Functional enrichment analysis of genes associated with these clusters further supported this interpretation (Fig. 4i). The most significantly enriched pathway was protein processing in the endoplasmic reticulum, reflecting the intense antibody synthesis activity characteristic of plasma cells. Additional enrichment of immune-related pathways, such as complement and coagulation cascades, pertussis, Staphylococcus aureus infection, and systemic lupus erythematosus, indicates that this region represents an active immune effector niche. Finally, examination of spatially variable genes located near the periphery of the germinal center showed that several genes with blurred spatial patterns in the original data became structurally well defined after Bell enhancement (Fig. 4k). These results indicate that Bell not only improves spatial resolution but also reveals previously hidden microstructural organization within tissues. To further support the biological interpretation of Bell-recovered microdomains, an independent colorectal cancer validation with marker inspection, Cell2Location deconvolution, and CellChat analysis is shown in Figs. S6 and S7 (Validation of Bell-Recovered Ecological Niches in Human Colorectal Cancer). Taken together, these analyses demonstrate a consistent progression: low-resolution measurements obscure tissue structure, whereas Bell reconstruction restores spatial continuity and reveals new microdomains. By recovering latent spatial structures that are masked by noise and spatial averaging, Bell enables the discovery of biologically meaningful tissue microenvironments that would be difficult to detect using raw spatial omics data alone.

### 3.5 mmBell can integrate spatial multi-omics to identify spatial domains and enhance biological patterns

**Figure 5:**
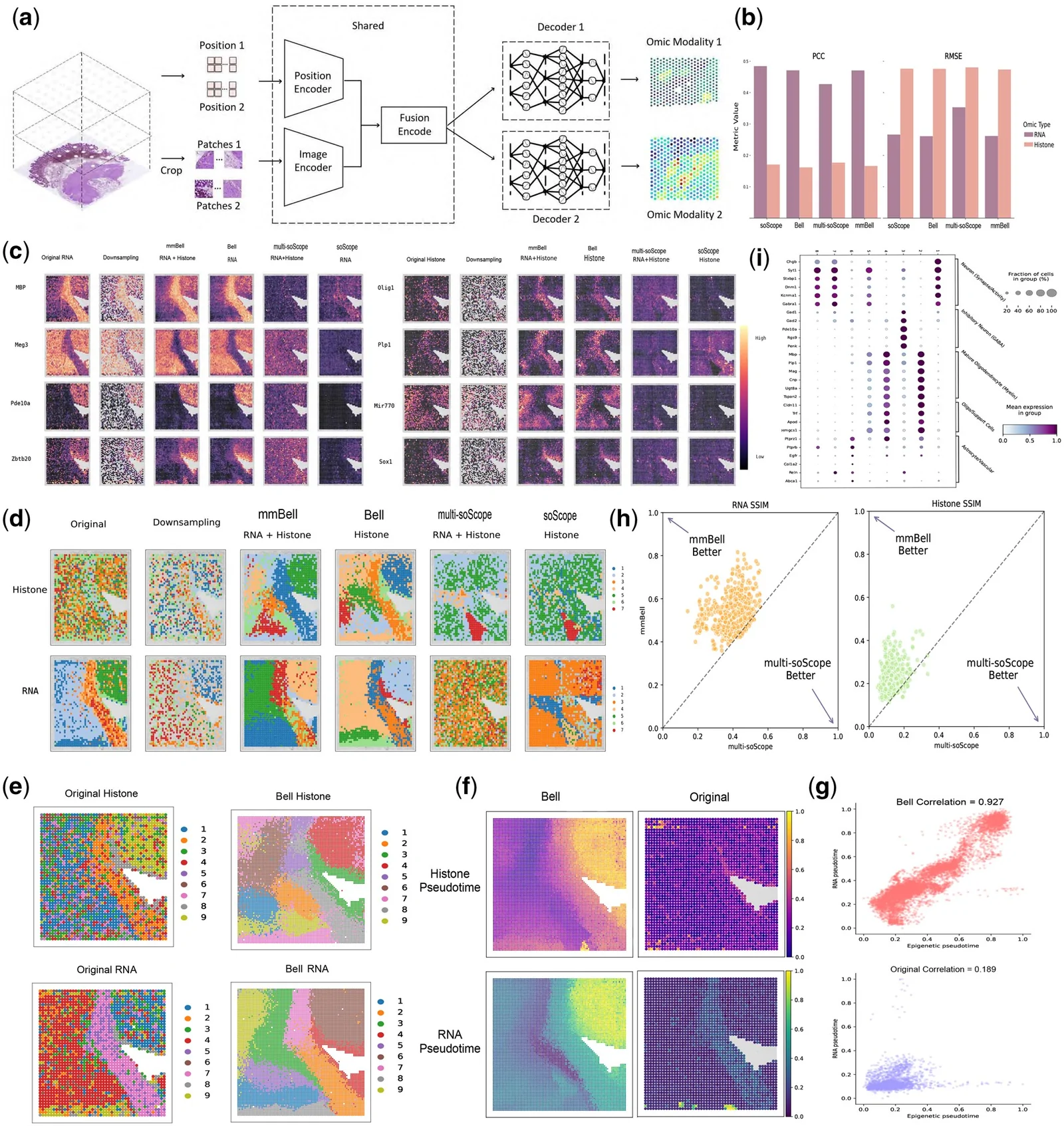
Application of Bell for multimodal spatial resolution enhancement. (a) The workflow of mmBell, which integrates image and spatial location features through a shared encoder and fusion encoding, while using independent decoders for reconstruction across different modalities. (b) Comparison of Pearson correlation coefficient (PCC) and root mean squared error (RMSE) between single-modality and multimodal scenarios on mouse brain tissue data generated by spatial-CUT&Tag-RNA-seq (Histone RNA ME). (c) Visualization comparison of spatially variable genes under single-modality and multimodal conditions on mouse brain tissue data. (d) Clustering comparison between original data and Bell-reconstructed data under single-modality and multimodal conditions on mouse brain tissue data. (e) Results of Bell 4× and direct clustering for histone and RNA data in mouse embryos. (f) Pseudotemporal analysis plots for Bell 4× and original data. (g) Correlation of histone and RNA data for Bell 4× and original data. (h) SSIM of histone and RNA data for mmBell and multi-soScope. (i) Differential analysis of mmBell clustering results.

Spatial multi-omics technologies provide complementary molecular-layer information that can substantially enhance the understanding of tissue architecture. However, as highlighted in the Introduction, multimodal spatial datasets are often affected by severe noise, sparsity, and modality imbalance. These factors obscure true biological signals and make cross-modality analyses—such as clustering and trajectory inference—unstable. To address these challenges, we extended the Bell framework to support multimodal spatial data integration and developed mmBell, a unified model designed to enhance spatial inference through joint multimodal learning. The architecture of mmBell adopts a shared encoder and fused representations to integrate positional information and image-derived features across different molecular modalities (Fig. 5a). These shared embeddings capture global tissue architecture and local spatial dependencies, while modality-specific decoders reconstruct enhanced molecular profiles for each modality. The entire system is trained end-to-end, allowing information from different omics layers to mutually regularize the reconstruction process. Compared with existing multimodal spatial enhancement approaches, this design enables mmBell to leverage cross-modality signals to stabilize spatial pattern recovery and reduce technical noise.

We first evaluated mmBell on a spatial CUT&Tag–RNA-seq dataset from mouse brain tissue, which simultaneously profiles histone modifications and transcriptomes across 2013 spatially matched spots. To simulate data loss and assess recovery capability, we downsampled the data from both modalities and attempted to reconstruct the missing components. Quantitative evaluation using PCC (Pearson correlation coefficient) and RMSE (root mean squared error) (Fig. 5b) showed that the single-modality Bell and soScope (Li *et al.* 2024a) exhibited comparable performance when reconstructing individual modalities. However, in the multimodal setting, mmBell demonstrated significantly more stable performance than multi-soScope, indicating that its integration strategy more effectively exploits complementary information across modalities. To further investigate how multimodal learning influences spatial pattern recovery, we examined the top 20 spatially variable genes (Fig. 5c). In the raw dataset, histone modification signals were considerably noisier than RNA expression and exhibited blurred spatial patterns. Under these high-noise conditions, the reconstruction quality of multi-soScope deteriorated markedly, whereas Bell maintained relatively stable performance. Importantly, multimodal integration in mmBell resulted in clearer and more coherent spatial patterns, suggesting that cross-modality information can effectively suppress noise and stabilize signal reconstruction. Clustering analysis further supported this observation (Fig. 5d). The original histone dataset was difficult to cluster due to excessive noise, while RNA-based clustering, although separable, remained unstable. After enhancement by Bell, the clustering structures of both modalities improved. Notably, mmBell produced substantially clearer and more consistent clusters than single-modality enhancement, indicating that multimodal integration helps reduce noise and improves spatial domain identification. A comparison of structural similarity between reconstructed and original datasets (Fig. 5h) confirmed that mmBell consistently achieved higher structural fidelity than multi-soScope.

Another advantage of mmBell lies in its flexible multimodal integration strategy. Existing methods such as multi-soScope concatenate features from different modalities and therefore require perfectly aligned spatial spots across modalities. In contrast, mmBell learns a unified spatial representation through a shared encoder, allowing the framework to integrate multimodal data even when measurements are obtained from different but overlapping spatial grids. We validated this capability using a human tonsil dataset generated by Visium CytAssist, which contains 4992 spatial proteomics spots and 4908 spatial transcriptomics spots. In this dataset, mmBell demonstrated significant improvements in both PCC and RMSE compared with single-modality models (Fig. S4c). Reconstruction examples (Fig. S4a) and clustering results (Fig. S4b) further confirmed the robustness of mmBell in recovering high-quality multimodal spatial profiles. Beyond improving signal quality, multimodal integration also enhances biological spatial inference. We applied mmBell to enhance the mouse brain dataset to fourfold spatial resolution, which revealed substantially clearer anatomical structures (Fig. 5e). Analysis of marker genes from RNA clusters derived from the mmBell-enhanced data (Fig. 5i) identified several biologically meaningful cell populations. Clusters 2 and 4 were enriched for myelination-related genes and correspond to terminally differentiated mature oligodendrocytes within the oligodendrocyte lineage. Clusters 1, 3, 7, and 8 represent neuronal populations with distinct subtype characteristics. Cluster 3, marked by Gad1/2 expression, corresponds to the lineage of GABAergic inhibitory neurons, whereas clusters 1, 7, and 8 express markers such as Chgb, Syt1, and Stxbp1, indicating excitatory or non-GABAergic neuronal subtypes. Cluster 6 is characterized by genes including Ptprz1, Egfr, and Col1a2, suggesting associations with astrocytes as well as vascular or endothelial cell populations. Importantly, the denoising and multimodal integration provided by mmBell substantially improved developmental trajectory inference. Pseudotime analyses performed separately on the raw dataset showed poor concordance between RNA-based and histone-based trajectories, reflecting the strong influence of technical noise (PCC = 0.189) (Fig. 5f and g). The trajectory-inference workflow used Scanpy Diffusion Pseudotime, with preprocessing, neighbor-graph construction, root-cell selection, and cross-modality comparison described in the Supplementary Materials. In contrast, trajectories inferred from the mmBell-enhanced data exhibited high cross-modality consistency (PCC = 0.927) and revealed a clear differentiation path from cluster 3 toward clusters 1, 7, and 8. This trajectory captures the progressive specialization and maturation of neuronal subtypes in the mouse brain, consistent with previous studies (Kirmse and Zhang 2022, Keefe *et al.* 2025). These results indicate that multimodal integration combined with effective noise suppression enables more reliable spatial developmental inference. Overall, these results demonstrate that mmBell enhances spatial inference through multimodal integration. By jointly reducing noise, stabilizing spatial patterns, and aligning biological signals across molecular layers, mmBell enables more accurate identification of spatial domains and more consistent developmental trajectories. These capabilities directly address the challenges outlined in the Introduction—namely, recovering hidden spatial structures from noisy and sparse spatial omics data—and highlight the potential of multimodal spatial learning for revealing fine-scale tissue organization.

## 4 Discussion

Although spatial omics has greatly advanced the analysis of tissue microarchitecture, it remains constrained by resolution limitations, noise, and data sparsity. In this study, the proposed Bell framework and its multimodal extension mmBell effectively overcome these limitations through image-guided deep learning. Systematic evaluations demonstrate that Bell not only significantly improves the reconstruction accuracy of single-gene expression but also preserves higher-order structures such as gene correlations and spatial topology, thereby providing a robust foundation for downstream analyses including regulatory network inference and cell–cell interaction analysis. One of the key strengths of this framework lies in its strong cross-modality generalization capability. Even in emerging platforms characterized by high noise, such as spatial DNA methylation, Bell can stably recover spatial structural signals. More importantly, Bell overcomes the spatial averaging effect inherent in conventional technologies and accurately reconstructs previously obscured tissue microenvironments.

To address the growing trend toward spatial multi-omics, we further propose mmBell, which integrates signals from different omics layers through a shared encoder architecture. This design enables mutual regularization and synergistic enhancement across modalities, substantially improving the consistency of spatial domain identification and cross-modal developmental trajectory inference. Although the performance of this framework partly depends on the quality and staining consistency of histological images, and there remains room for computational efficiency optimization when handling ultra-large datasets at the million-spot scale, overall Bell and mmBell provide a unified and scalable solution. In particular, staining batch effects, scanner or illumination differences, image compression, and local artifacts such as tissue folds, tears, out-of-focus regions, or uneven staining may affect image-derived features and introduce biases that are absent from purely molecular super-resolution methods. Because histological morphology does not always uniquely determine molecular state, excessive image dependence may overemphasize morphology-associated boundaries or underrepresent molecular programs with weak histological signatures. Bell mitigates this risk by jointly integrating image features, spatial coordinates, and observed molecular profiles through adaptive attention, rather than relying on image information alone. Bell has not yet been evaluated using fully controlled synthetic spatial transcriptomics simulations. Although simulations such as SynthSpot provide known cell-type compositions and artificial tissue regions, they do not include paired histological images or realistic morphology–molecular relationships, which are core inputs for Bell. Therefore, they cannot be directly used to evaluate the complete image-guided framework without simplifying or disabling the image component. Nevertheless, such simulations remain valuable complementary benchmarks. Future evaluations should test Bell under additional controlled scenarios with known spatial domains, known cell-type mixtures, and tunable noise, sparsity, and morphology–expression coupling. These settings would further assess its robustness and its tendency to avoid generating artificial microstructures. In practical applications, tissue/background masking, artifact filtering, color normalization, stain augmentation, image-ablated or molecular-only controls, and validation with independent biomarkers or orthogonal modalities can further improve robustness. By deeply integrating imaging data, spatial coordinates, and multi-omics information, Bell and mmBell successfully reconstruct high-fidelity spatial molecular landscapes and are expected to play a critical role in future studies of complex tissues, including tumor biology, developmental biology, and immune microenvironments.

## Supplementary Material

btag596_Supplementary_Data

## Data Availability

Bell source code is publicly available from the GitHub repository. An archived, citable software snapshot has been deposited on Zenodo. All datasets in this study are publicly available. The raw datasets are available as following: HI: https://data.mendeley.com/datasets/v8s9nz948s/1 HSCC: https://data.mendeley.com/datasets/2bh5fchcv6/1 DLPFC: http://research.libd.org/spatialLIBD OF: https://osf.io/mqsb4/? view_only= BC: GSE213688 TLS: GSE175540 MB: https://github.com/STOmics/Stereopy ME: https://db.cngb.org/stomics/datasets/STDS0000075/data MS: GSE198353 HBC: https://www.10xgenomics.com/products/xenium-in-situ/preview-dataset-human-breast HD HT: https://cf.10xgenomics.com/samples/spatial-exp/3.1.2/Visium_HD_FF_Human_Tonsil HD HLC: https://cf.10xgenomics.com/samples/spatial-exp/3.0.0/Visium_HD_Human_Lung_Cancer_HD_Only_Experiment1 Protein HTA: https://www.10xgenomics.com/datasets Protein HT: https://www.10xgenomics.com/datasets Protein HBC: https://www.10xgenomics.com/datasets Protein MS: GSE198353 Metabolomics MB: https://upenn.app.box.com/s/3o8dq5j4x29ic6zo7iugdo83scnv4qis Histone ME: GSE165217 Histone RNA ME: GSE205051, GSE205054 ATAC ME: GSM6801813 Spatial-DMT: GSE270498 Human colorectal cancer spatial transcriptomics: 10x Genomics human colorectal cancer spatial transcriptomics dataset Human colorectal cancer single-cell transcriptomics: GSE144735.
