# Supplementary material for: Image-guided spatial omics enhancement reveals hidden spatial microstructures
Source: Bioinformatics. 2026 Aug 7;42(8):btag596. doi: 10.1093/bioinformatics/btag596 (PMC13485349; doi:10.1093/bioinformatics/btag596)
Supplement: Supplementary file 1 [file btag596_supplementary_data.pdf]

Supplementary Materials for Image-guided Spatial Omics Enhancement Reveals Hidden Spatial Microstructures

Jiahao Liu[1], Gongning Luo[2], Qiaoming Liu[3], Suyu Dong[1], Guohua Wang*[1,2], and Yuming Zhao*[1]

[1]College of Computer and Control Engineering, Northeast Forestry University, Heilongjiang, China
[2]Faculty of Computing, Harbin Institute of Technology, Heilongjiang, China
[3]College of Artificial Intelligence, Henan University, Henan, China

Table S1: Summary of datasets. Abbr is the abbreviation of the corresponding dataset in the text.

Abbr	Dataset Name	Data Type	Platform
HI	Human Ileum	Spatial Transcriptomics	Visium
HSCC	Human Squamous Cell Carcinoma	Spatial Transcriptomics	Visium
DLPFC	Human Dorsolateral Pre-Frontal Cortex	Spatial Transcriptomics	Visium
OF	Orchid Flowers Organogenesis	Spatial Transcriptomics	Visium
BC	Breast Cancer Sub-types	Spatial Transcriptomics	Visium
TLS	Tertiary Lymphoid Structures	Spatial Transcriptomics	Visium
MB	Mouse Brain	Spatial Transcriptomics	Stereo-Seq
ME	Mouse Embryos	Spatial Transcriptomics	DBiT-seq
MS	Mouse Spleen	Spatial Transcriptomics	Visium
HBC	Human Breast Cancer	Spatial Transcriptomics	Xenium
HD HT	Human Tonsil	Spatial Transcriptomics	HD Visium
HD HLC	Human Lung Cancer	Spatial Transcriptomics	HD Visium
Protein HTA	Human_Tonsil	Spatial Transcriptomics, Spatial Proteomics	Visium CytAssist
Protein HT	Human Tonsil	Spatial Transcriptomics, Spatial Proteomics	Visium CytAssist
Protein HBC	Human Breast Cancer	Spatial Transcriptomics, Spatial Proteomics	Visium CytAssist
Protein MS	Mouse Spleen	Spatial Transcriptomics, Spatial Proteomics	SPOT
Metabolomics MB	Mouse Brain	Spatial Transcriptomics, Spatial Metabolomics	MALDI-MSI, Visium
Histone ME	Mouse Embryo	Spatial Epigenomics	Spatial-CUT&Tag
Histone RNA ME	Mouse Embryo	Spatial Epigenomics, Spatial Transcriptomics	spatial-CUT&Tag-RNA-seq
ATAC ME	Mouse Embryo	Spatial Epigenomics	ATAC-Seq

Table S2: Computational resource consumption of Bell at representative input scales. This table reports the primary CPU and GPU memory measurements at 2,379, 9,124, and 47,510 spots. Together with the dataset-wise GPU scaling curve in Supplementary Fig. S3, these measurements define the resource-analysis results and are distinct from the dataset-specific runtime subsets shown in Fig. 2e. Hardware: Intel Xeon Platinum 8358 CPU and NVIDIA RTX 4090 GPU.

**Spot Number**	**CPU Memory (GB)**	**GPU Memory (GB)**
2379	6.26	0.36
9124	7.74	0.96
47510	7.67	4.70

Table S3: Architecture and training settings of Bell and mmBell. The settings summarize the model description and default training configuration used in this study.

**Component or setting**	**Bell**	**mmBell**
Input data	Histological image, spatial coordinates, and one spatial molecular profile.	Histological image, spatial coordinates, and molecular profiles from two or more spatial omics modalities.
Molecular feature preprocessing	Mitochondrial genes are removed; genes expressed in fewer than 30 spots are filtered out; Moran's I is calculated for remaining genes; Moran's I values are clustered with DBSCAN; approximately 30% of genes are sampled from each Moran's I cluster to preserve diverse spatial patterns. For uniform benchmark preprocessing, samples with more than 3000 features are further represented by the top 3000 highly variable features; otherwise, all features are retained.	The same molecular preprocessing is applied independently to each modality when applicable. For multimodal data such as CUT&Tag, ATAC-seq, proteomics, or metabolomics, modality-specific molecular matrices are first converted into spot-by-feature matrices and then processed with the same normalization and feature-selection workflow where applicable.
Normalization	Molecular profiles are normalized using `normalize_total` and `log1p`.	Each modality is normalized independently using the same strategy where applicable, reducing scale differences before joint training.
Image patch input	A 32×32 image patch centered on each spatial spot is used as the visual input.	The same image-patch strategy is used for each modality-specific spatial location or overlapping spatial grid.
Image encoder	A pretrained ResNet18 extracts 512-dimensional image embeddings, which are projected to 128 dimensions using a linear layer with Dropout.	A shared image encoder is used to process image patches across modalities.
Spatial-position encoder	The x and y pixel coordinates of each spot are used as positional inputs and projected to a 128-dimensional representation through a KAN layer with Batch Normalization.	A shared position encoder is used to process modality-specific spatial coordinates. This design allows mmBell to integrate modalities measured on different but overlapping spatial grids.

Continued on next page

Table S3 – continued from previous page

Component or setting	Bell	mmBell
Feature fusion	A 128-dimensional adaptive attention mechanism weights image features and fuses them with spatial-position embeddings. The attention vector is initialized to ones and jointly optimized during training.	The shared fusion module integrates image and positional representations across modalities before modality-specific reconstruction.
Fusion encoder	Two stacked KAN layers learn the fused image-position representation. A residual branch directly passes the spatial-position latent representation into the fused encoding to reduce spatial-information loss.	The same shared fusion encoder is used for all modalities.
Decoder	Four stacked KAN layers reconstruct the molecular profile. The first three KAN layers keep a 128-dimensional feature space, and the final KAN layer maps the representation to the target molecular-feature dimension.	Independent modality-specific decoders reconstruct each molecular modality separately from the shared fused representation.
Loss function	Mean squared reconstruction error between predicted and observed molecular profiles, $\ell_{Bell} = \frac{1}{n}\sum_{j=1}^{n}(v_j - \hat{v}_j)^2$.	Sum of modality-specific mean squared reconstruction errors, $\ell_{mmBell} = \frac{1}{n}\sum_{j=1}^{n}(v_j^1 - \hat{v}_j^1)^2 + \frac{1}{m}\sum_{j=1}^{m}(v_j^2 - \hat{v}_j^2)^2$ for the two-modality case.
Training mode	Self-supervised training on each sample by minimizing reconstruction error between predicted and observed molecular profiles.	End-to-end joint training of shared encoders/fusion module and independent decoders.
Dynamic sampling strategy	During the first 90% of training epochs, 20%–40% of all spots are dynamically sampled for training; during the final 10%, all spots are used for fine-tuning. Spatial locations are randomly shuffled during training.	The same dynamic sampling and fine-tuning strategy is used, with modality-specific losses jointly optimized.
Default optimizer and learning rate	Adam optimizer with learning rate 0.001.	Adam optimizer with learning rate 0.001.

Continued on next page

Table S3 – continued from previous page

**Component or setting**	**Bell**	**mmBell**
Default training epochs	500 epochs for recovery tasks and 3000 epochs for high-resolution generation.	The same default epoch settings are used unless otherwise specified for a dataset.
Default sensitivity-analysis settings	Epoch = 3000, batch size = 10000, patch radius = 16, and dynamic sampling range = 0.2–0.4.	The same settings provide the default reference configuration for multimodal experiments, with independent decoders for each modality.
High-resolution grid generation	New candidate spots are generated on a grid at an N-fold higher density. Grid points are retained only when their minimum distance to the original spatial positions is no greater than the average nearest-neighbor distance among original spots, preserving the overall tissue shape.	The same high-resolution grid strategy is used when generating enhanced multimodal molecular maps.

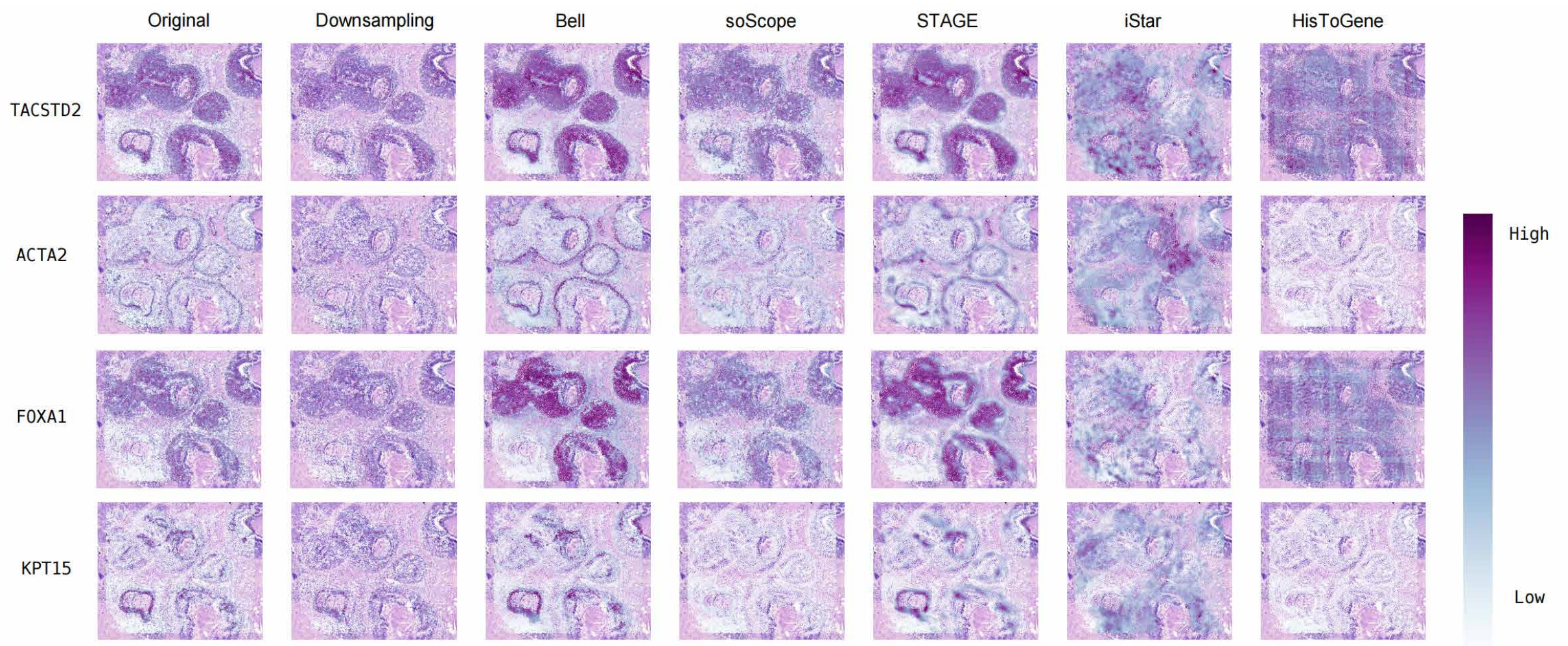

Figure S1: **ROI selection and marker-gene reconstruction in a human breast cancer (HBC) Xenium dataset.** A predefined ROI from the large whole-section dataset was selected before model reconstruction according to tissue coverage, spot density, image quality, and tumor-boundary structure. Spatial patterns of representative marker genes are shown for the original data, downsampled input, Bell, and competing reconstruction methods.

Hyperparameter Sensitivity Analysis and Training Settings

To evaluate the robustness of Bell under different training settings, we performed a systematic hyperparameter sensitivity analysis. We used a single-factor controlled experimental design: in each experiment, only one hyperparameter was changed, while all other parameters were fixed at the default setting. The default parameters were epoch = 3000, batch size = 10000, patch radius = 16, and dynamic sampling range = 0.2–0.4.

The analyzed hyperparameters included the number of training epochs, batch size, spatial patch radius, and dynamic sampling range. Specifically, epoch was set to 500, 1000, 1500, 2000, 2500, and 3000 to evaluate whether model performance continued to improve with longer training or converged under fewer iterations. Batch size was set to 1024, 2048, 4096, 8000, 10000, and 16000 to examine its effect on reconstruction stability, training efficiency, and memory usage. Patch radius was set to 16, 24, 32, and 48 to evaluate how different spatial neighborhood sizes affected the use of local spatial context. Dynamic sampling range was set to 0.1–0.2, 0.2–0.4, 0.3–0.6, 0.5–0.8, 0.8–1.0, and full, where full indicates training with the complete batch without dynamic sampling.

The sensitivity analysis was conducted on three representative spatial transcriptomics datasets, including CytAssist Human Tonsil, Visium osfstorage, and Stereo-seq mouse brain. We evaluated PCC, SSIM, and RMSE, together with changes in training-resource usage. Overall, changing the random seed caused negligible performance fluctuation, indicating that Bell training is stable. Batch size had little effect on reconstruction quality, which is consistent with the full-gene random sampling and perturbation strategy used during optimization. As the number of epochs increased, PCC and RMSE showed slight decreases in some datasets after prolonged training, suggesting a mild overfitting tendency under excessive training. However, the overall performance remained relatively stable. Increasing patch radius generally improved reconstruction, because larger patches provide more spatial context for matching local expression patterns.

The dynamic sampling range mainly determines the proportion and difficulty range of spatial points or patches used in each training iteration. A smaller sampling range makes the task more difficult, because the model must recover expression from less observed spatial information. This acts as a stronger perturbation and can improve generalization. A larger sampling range makes the task easier, because the model can access more observed information, but it may increase the risk of rapid overfitting. Since typical spatial omics datasets contain thousands to tens of thousands of molecular features, we recommend using a relatively small dynamic sampling range so that Bell learns robust spatial context instead of rapidly overfitting to easier reconstruction tasks.

Trajectory Inference for Multimodal Pseudotime Analysis

For the pseudotime analysis shown in the multimodal mouse brain experiment, we used Diffusion Pseudotime (DPT) implemented in Scanpy. The analysis was performed separately for the spatial RNA expression matrix and the CUT&Tag GeneScoreMatrix. For each modality, spots were treated as individual observations, and their molecular profiles were used to define similarity among spots.

Briefly, each matrix was first normalized and reduced by principal component analysis

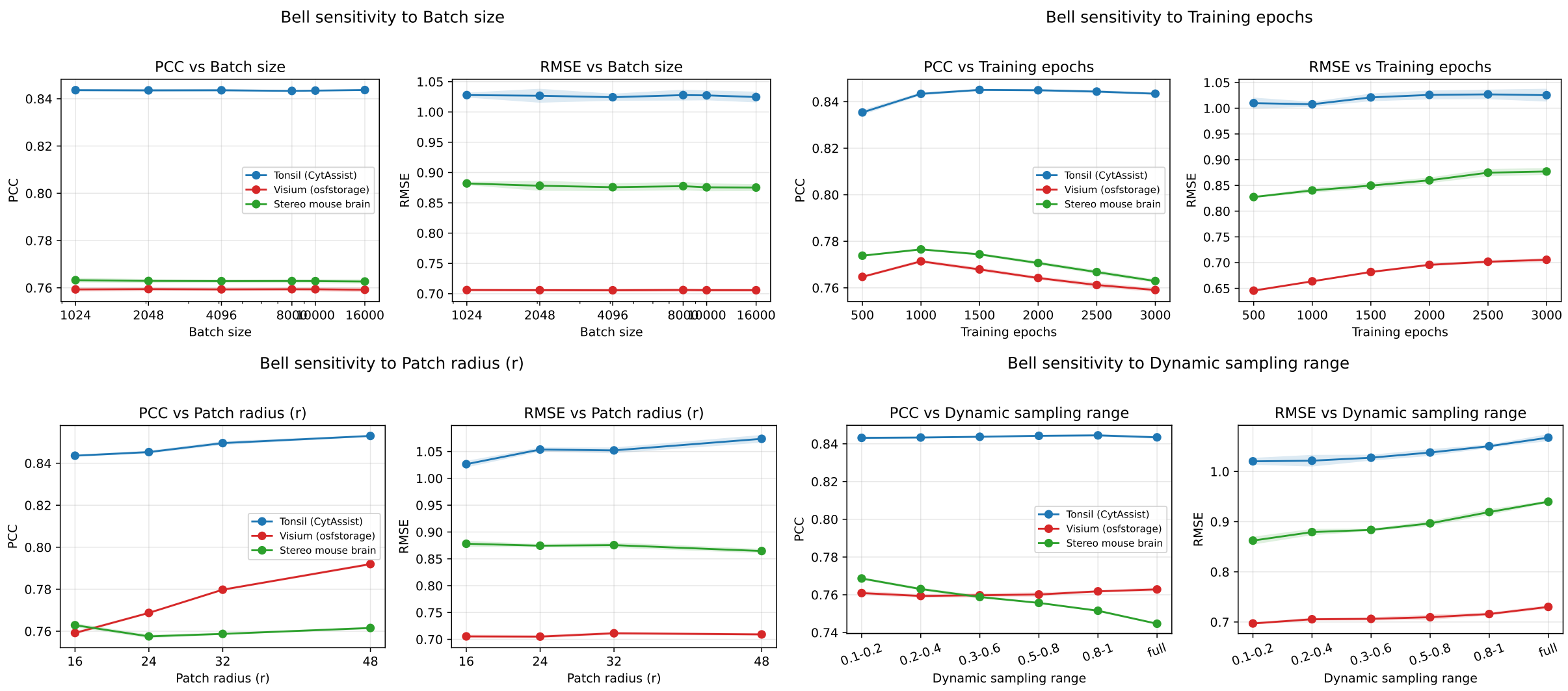

Figure S2: **Hyperparameter sensitivity analysis of Bell.** PCC and RMSE were evaluated under single-factor changes in batch size, training epoch number, spatial patch radius, and dynamic sampling range. The analysis was performed on three representative spatial transcriptomics datasets: CytAssist human tonsil, Visium osfstorage, and Stereo-seq mouse brain.

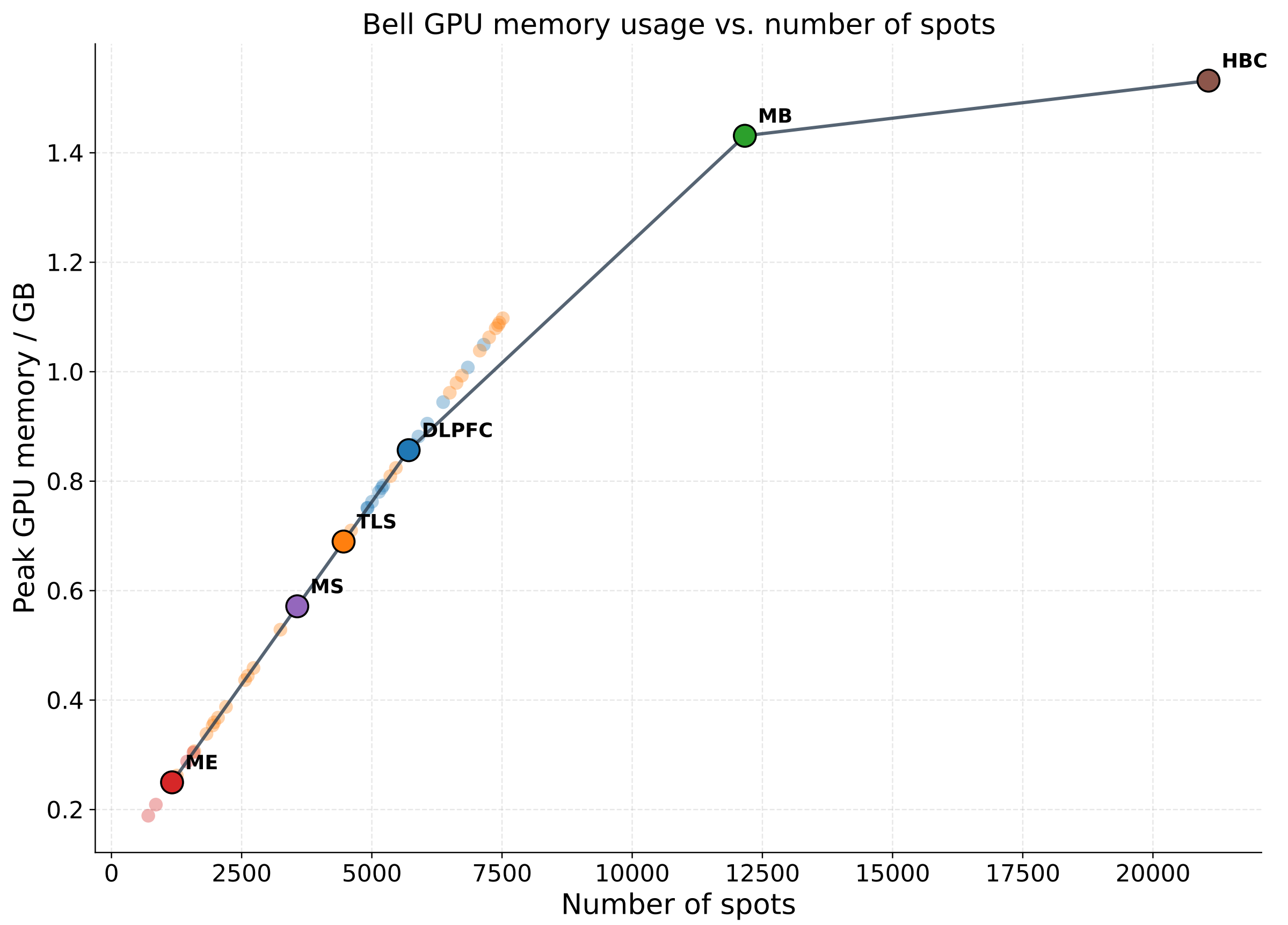

Figure S3: **GPU memory usage of Bell across datasets with different spot numbers.** Peak GPU memory usage is shown as a function of the number of spatial spots for ME, MS, TLS, DLPFC, MB, and HBC datasets. The curve indicates that Bell maintains low GPU memory demand and scales stably as the input spot number increases.

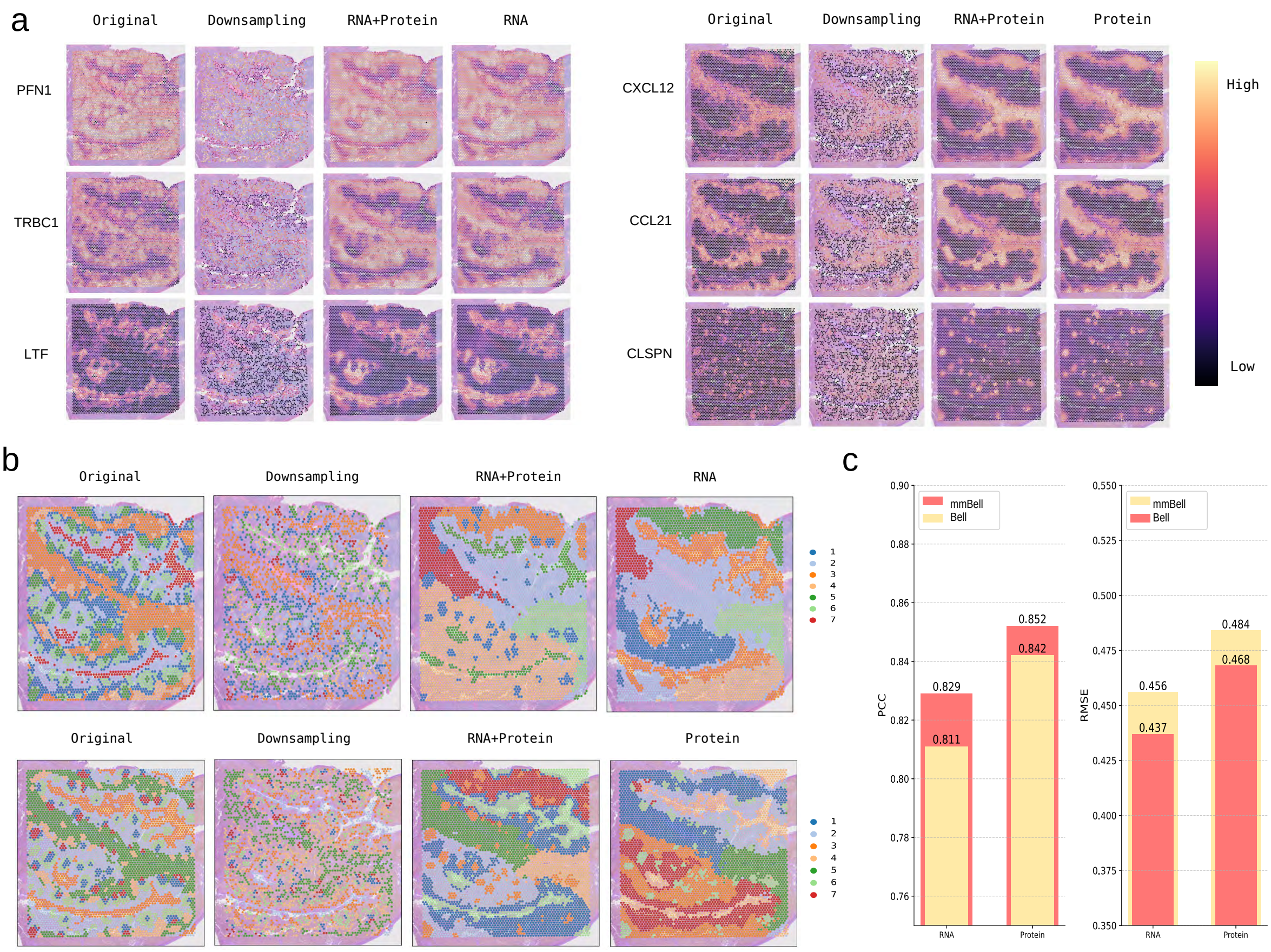

Figure S4: **mmBell multimodal integration on human tonsil spatial proteomics and transcriptomics data (Protein HTA). a** Reconstruction results of spatially variable genes by Bell when spot distributions of the two modalities are misaligned. **b** Single-modality vs. multimodal comparison of Bell on human tonsil data. **c** Comparison of PCC and RMSE for reconstruction results between single-modality Bell and multimodal mmBell on human tonsil data.

(PCA) to retain the dominant sources of molecular variation. A k-nearest-neighbor graph was then constructed using `sc.pp.neighbors`, followed by diffusion map calculation with `sc.tl.diffmap`. The diffusion map was used to represent the continuous manifold structure of the spatial molecular states. We selected the spot with the largest value on the first diffusion component as the root cell and then computed diffusion pseudotime using `sc.tl.dpt`. The resulting pseudotime values represent the relative ordering of spots along a continuous biological state transition, rather than a strict branched lineage reconstruction.

RNA-based pseudotime was inferred from transcriptional expression variation, whereas histone-based pseudotime was inferred from epigenomic GeneScore variation. The two pseudotime vectors were subsequently compared by correlation analysis and visualized in both scatter and spatial plots. This analysis was used to evaluate whether the transcriptional and epigenomic trajectories were concordant after mmBell enhancement and whether the two modalities showed consistent spatial organization during the inferred state transition.

Visium HD Ground-Truth Benchmark

Following the realistic-degradation evaluation framework used in spatial-omics benchmarking studies such as Spotless (Sang-aram et al. 2024), we designed a benchmark based on the human tonsil Visium HD dataset. In this benchmark, high-resolution bin-level expression from a germinal-center candidate ROI in the Visium HD human tonsil R3 sample was used as the reference ground truth. We first selected highly variable genes and retained a set of biologically interpretable tonsil marker genes, including CD3D, MS4A1, and CXCL13, so that the evaluation reflected both statistical reconstruction accuracy and biologically meaningful tissue organization.

To generate a realistic low-resolution input, the original high-resolution expression matrix was aggregated into lower-resolution spots using a fixed spatial grid. We then added multiple sources of sequencing-like degradation to the aggregated counts, including library-size downsampling, negative-binomial overdispersion, dropout, and ambient RNA contamination. This procedure produced a degraded low-resolution input that better approximates noisy, lower-quality spatial transcriptomics measurements than simple spatial averaging alone. All evaluated methods were trained and tested using only this degraded low-resolution input and the target high-resolution coordinates. Predictions from all methods were compared against the original high-resolution Visium HD measurements on the same normalized scale after `normalize_total` and `log1p` transformation.

The evaluation metrics included gene-wise spatial correlation and error, spot-wise expression-profile correlation and error, and global RMSE. This design assesses whether each method recovers spatial gene-expression patterns at the gene level and whether it reconstructs the full expression profile at each spatial location. It also reduces the risk that a method could appear favorable under a single metric through oversmoothing or other trivial prediction strategies. Because the high-resolution target was derived from real Visium HD measurements rather than synthetic expression values, and because the degradation process was applied only to the low-resolution input, the benchmark avoids leakage of the high-resolution truth into the reconstruction model. All methods used the same ROI, gene set, degraded input, target coordinates, normalization procedure, and evaluation metrics, providing a controlled comparison in which performance differences are primarily attributable to the methods themselves.

In this benchmark, Bell consistently outperformed the competing methods in gene-wise PCC, spot-wise PCC, and RMSE (Fig. S5a). The original high-resolution truth showed clear tissue organization in the clustering and UMAP analyses, whereas the degraded low-resolution input showed substantially blurred structures after downsampling and noise addition (Fig. S5b). Under this challenging setting, several competing methods failed to recover the original high-resolution organization, whereas Bell reconstructed spatial structures that closely matched the high-resolution truth. Spatial inspection further showed that a region on the right side of the ROI was strongly disrupted after degradation, while Bell recovered a spatial pattern highly consistent with the original high-resolution data (Fig. S5c). These results support Bell's ability to recover biologically interpretable microstructures from noisy low-resolution spatial measurements under a ground-truth-like evaluation setting.

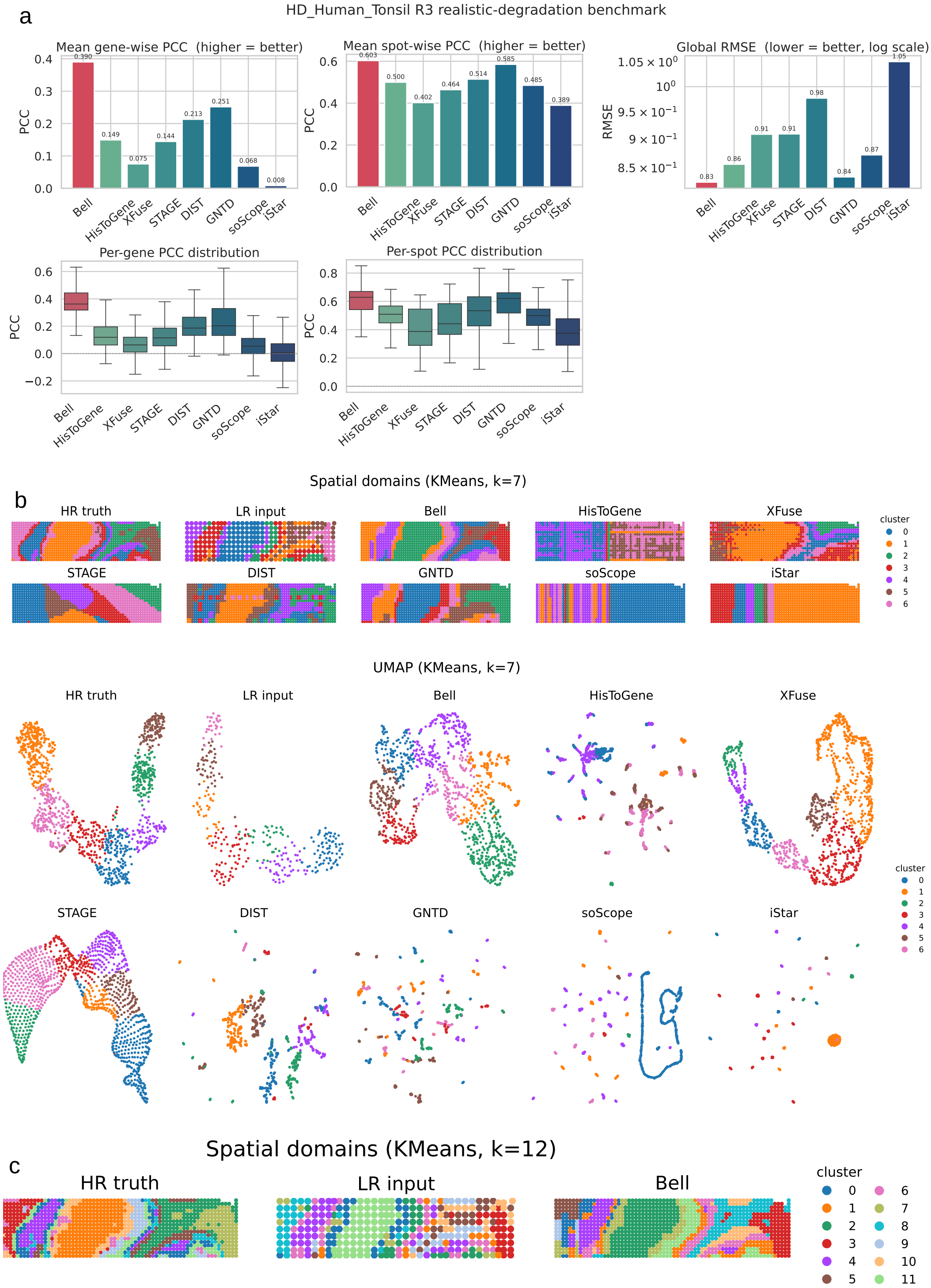

Figure S5: **Gold-standard-like benchmark using high-resolution Visium HD human tonsil data.** **a** Quantitative comparison of Bell and competing methods against the original high-resolution Visium HD ground truth using gene-wise PCC, spot-wise PCC, and RMSE. **b** Spatial-domain and UMAP comparison for the high-resolution truth, degraded low-resolution input, and method reconstructions using KMeans clustering with $k = 7$. **c** Spatial-domain comparison among the high-resolution truth, degraded low-resolution input, and Bell reconstruction under $k = 12$.

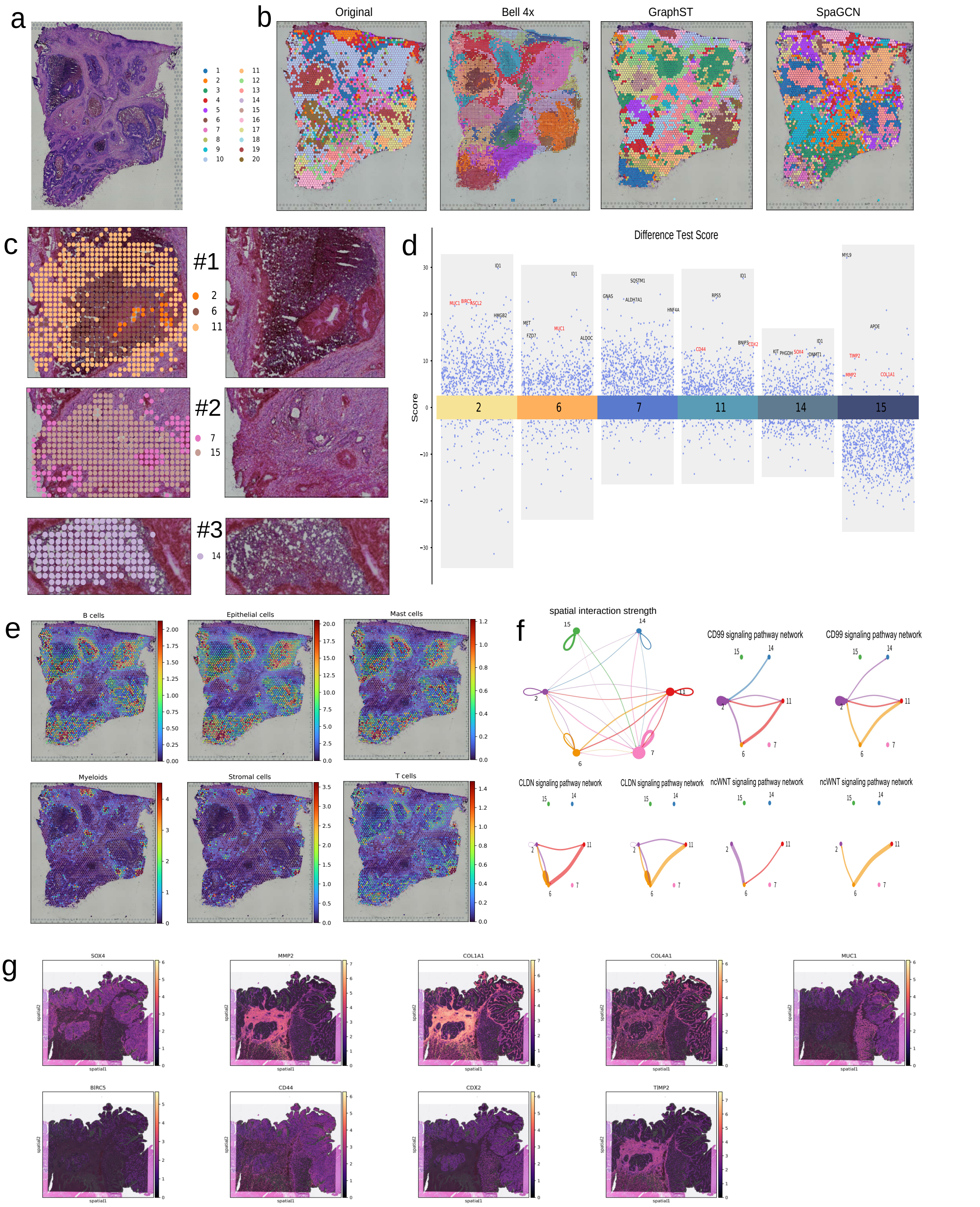

Figure S6: **Validation of Bell-recovered ecological niches in human colorectal cancer.** **a** H&E-stained tissue image of the analyzed colorectal cancer spatial transcriptomics section. **b** Spatial clustering comparison among the original data, Bell-enhanced data at 4× resolution, GraphST, and SpaGCN. **c** Bell-recovered ecological niche regions and corresponding pathological image regions. **d** Differentially expressed genes for Bell-defined clusters 2, 6, 7, 11, 14, and 15. **e** Cell2Location deconvolution of major cell types. **f** CellChat analysis of spatial interaction strength and representative signaling networks. **g** Spatial expression of representative marker genes in an independent high-resolution human colorectal cancer Visium HD dataset.

Validation of Bell-Recovered Ecological Niches in Human Colorectal Cancer

We used a human colorectal cancer spatial transcriptomics dataset to demonstrate the advantages of Bell in enhancing the identification and interpretation of spatial ecological niches. The dataset contains 3,138 spots (tissue image shown in Fig. S6a), and Bell was applied to increase the resolution to 4×. In the original data (Fig. S6b and Supplementary Fig.S7), although some relatively large tumor regions could be identified, many fine-grained details were not well resolved. We further compared Bell with several state-of-the-art clustering methods, including GraphST (Long et al. 2023), PROST (Liang et al. 2024), STAGATE (Dong and Zhang 2022), SEDR (Xu et al. 2024), and SpaGCN (Hu et al. 2021). In the results generated by GraphST, many ecological domains appeared fragmented, which may be attributed to the incomplete spatial graph structure in the complex tumor microenvironment. PROST, which is also based on a PNN framework, delineated relatively independent regions but failed to capture critical connections between different clusters. In contrast, after resolution enhancement by Bell, tissue details became markedly more prominent and clearer. We focused on several regions that are highly relevant to colorectal cancer (Regions #1, #2, and #3 in Fig. S6c). The Bell-enhanced clustering results showed a high degree of consistency with the pathological details observed in the tissue images. To validate the identities of these clusters, we performed differential expression analysis on the Bell clustering results (Fig. S6d). In region #1, clusters 2, 6, and 11 exhibited differentially expressed genes such as ASCL2, MUC1, ID1, BIRC5, CDX2, and CD44, indicating highly proliferative cells with strong epithelial markers and enrichment of metabolic- and stem cell–related genes. These features are characteristic of highly proliferative tumor epithelial cells and cancer stem cells, suggesting that region #1 corresponds to the tumor core and a highly proliferative zone. Compared with region #1, region #2 showed lower levels of cell proliferation and reduced expression of epithelial markers, while extracellular matrix (ECM) and stroma-related signals (e.g., COL1A1, COL4A1, MMP2, and TIMP2 in cluster 15) were enriched. This region represents the supportive structure surrounding the tumor or a stress-response area and can be defined as a tumor microenvironment and stromal remodeling zone. Region #3 exhibited intermediate characteristics between epithelial and stromal features, with enrichment of stemness-, stress-, and transcriptional regulation–related signatures. Notably, SOX4 and TGFBR2, which are closely associated with epithelial–mesenchymal transition (EMT) and the TGF-β signaling pathway, are typically observed at the invasive boundary where tumors infiltrate deeper tissues. Therefore, region #3 can be interpreted as the tumor front and an initiation zone of invasion. We further examined a high-resolution human colorectal cancer Visium HD dataset and found that these detected genes also showed corresponding morphological features around tumor regions (Fig. S6g). This result indicates that the spatial microstructures detected by Bell are present in the tissue and have biological relevance.

We next applied Cell2Location (Kleshchevnikov et al. 2022), using the single-cell dataset provided by Lee et al. (Lee et al. 2020) for deconvolution (Fig. S6e). The results showed that mast cells, B cells, and epithelial cells were enriched within the tumor regions, whereas T cells were mainly distributed around the tumor. Together, these cell types represent an active immune microenvironmental niche within the tumor. The spatial distribution of these cells was largely consistent with the ecological niches identified by Bell. Based on the ecological niches defined by Bell, we further applied CellChat (Jin

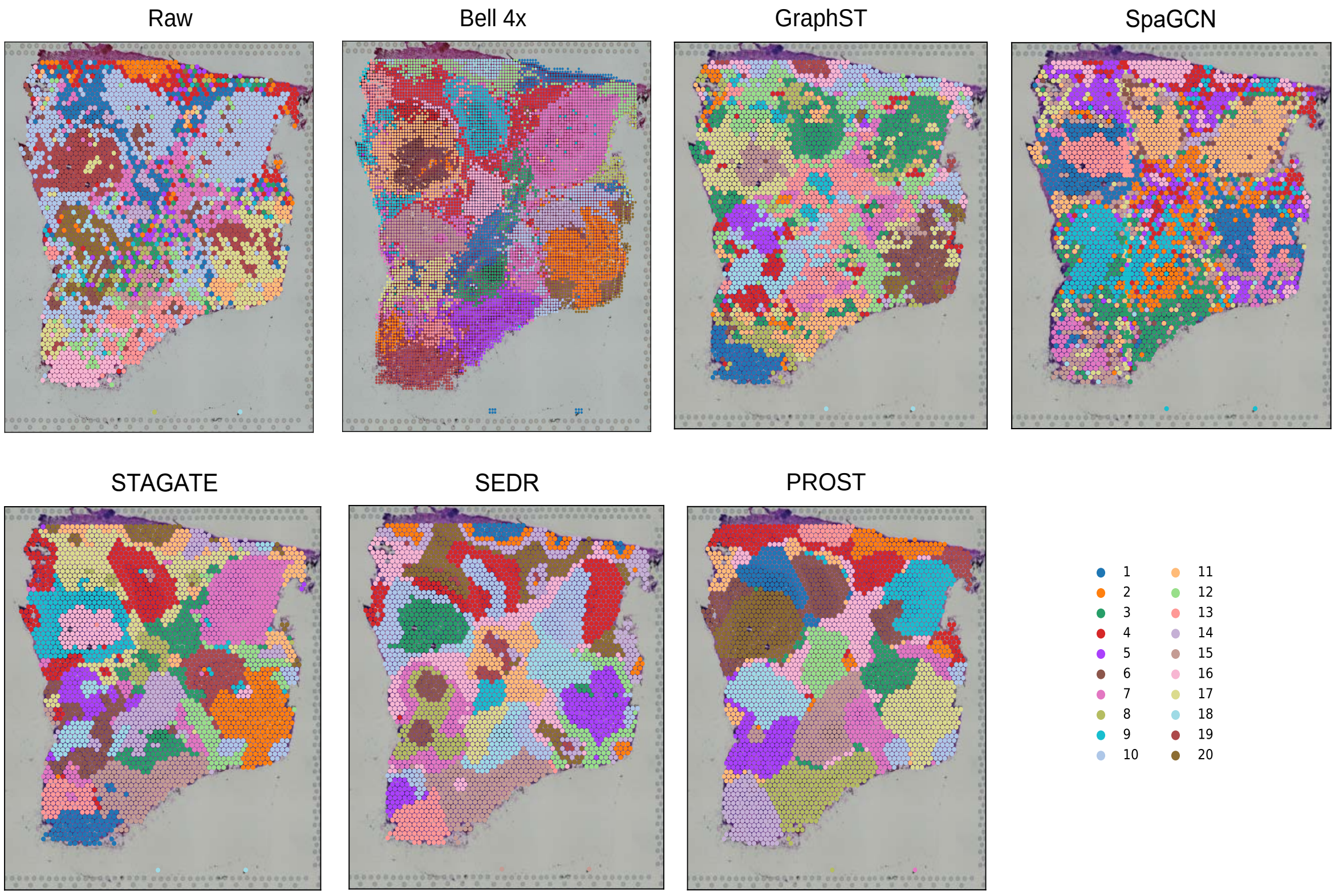

Figure S7: **Clustering comparison on the human colorectal cancer spatial transcriptomics dataset.** Spatial cluster assignments are shown for the raw data, Bell-enhanced data at 4× resolution, and results generated by GraphST, SpaGCN, STAGATE, SEDR, and PROST.

et al. 2021) to investigate epithelial–mesenchymal transition (EMT)-related cell–cell communication in colorectal cancer tissues (Fig. S6f). Analysis of spatial interaction strength revealed significant and tight interactions among clusters 2, 6, 7, and 11 within the tumor core. Among these, the CLDN and CD99 signaling pathways, which are closely associated with extracellular matrix (ECM) remodeling and disruption of cell adhesion, were predominantly active between clusters 2, 6, and 11. In contrast, the ncWNT pathway, known to promote EMT, maintain cellular stemness, and drive subpopulation differentiation, showed major communication between clusters 2 and 14. This suggests that tumor cells may be dissociating from the main tumor mass and infiltrating outward. Overall, these results demonstrate that Bell can effectively resolve tissue ecological niche heterogeneity at high spatial resolution and identify functionally and ecologically meaningful community types, thereby providing a powerful framework for understanding the spatial organization of functional states within the tissue microenvironment.

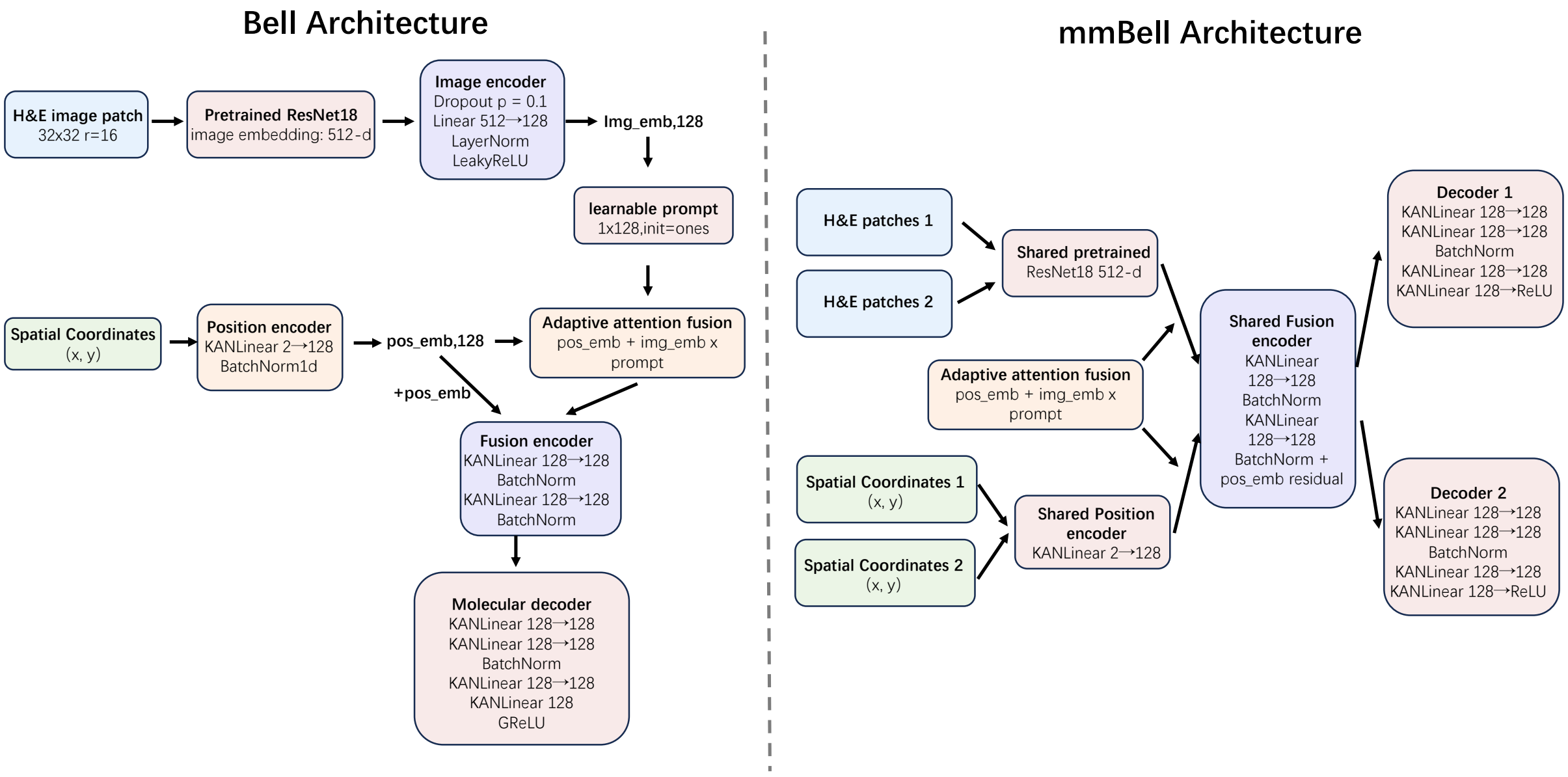

Figure S8: **Model architectures of Bell and mmBell.**

References

Dong K, Zhang S. Deciphering spatial domains from spatially resolved transcriptomics with an adaptive graph attention auto-encoder. *Nat Commun* 2022;13:1739.

Hu J et al. SpaGCN: integrating gene expression, spatial location and histology to identify spatial domains and spatially variable genes by graph convolutional network. *Nat Methods* 2021;18:1342–51.

Jin S et al. Inference and analysis of cell-cell communication using cellchat. *Nat Commun* 2021;12:1088.

Kleshchevnikov V et al. Cell2location maps fine-grained cell types in spatial transcriptomics. *Nat Biotechnol* 2022;40:661–71.

Lee HO et al. Lineage-dependent gene expression programs influence the immune landscape of colorectal cancer. *Nat Genet* 2020;52:594–603.

Liang Y et al. PROST: quantitative identification of spatially variable genes and domain detection in spatial transcriptomics. *Nat Commun* 2024;15:600.

Long Y et al. Spatially informed clustering, integration, and deconvolution of spatial transcriptomics with graphst. *Nat Commun* 2023;14:1155.

Sang-aram C et al. Spotless, a reproducible pipeline for benchmarking cell type deconvolution in spatial transcriptomics. *eLife* 2024;12:RP88431.

Xu H et al. Unsupervised spatially embedded deep representation of spatial transcriptomics. *Genome Med* 2024;16:12.
